# Removal of SARS-CoV-2 Antivirals from Aqueous Media Using Bioaugmented Activated Sludge

**DOI:** 10.3390/toxics14090786

**Published:** 2026-09-05

**Authors:** Dora Lastovčić, Ivona Zirn, Tijana Jezerčić, Kristina Bule Možar, Luka Večenaj, Matija Cvetnić, Marinko Markić, Marin Ganjto, Tomislav Bolanča, Dajana Kučić Grgić

**Affiliations:** 1 Faculty of Chemical Engineering and Technology, University of Zagreb, Trg Marka Marulića 19, 10000 Zagreb, Croatia; izirn@fkit.unizg.hr (I.Z.); tjezercic@fkit.unizg.hr (T.J.); lvecenaj@fkit.unizg.hr (L.V.); mcvetnic@fkit.unizg.hr (M.C.); mmarkic@fkit.unizg.hr (M.M.); tbolanca@unizg.hr (T.B.); 2 Central Waste Water Treatment Plant Zagreb, Heinzelova 70, 10000 Zagreb, Croatia; ganjto.marin@gmail.com

**Keywords:** SARS-CoV-2 antiviral substances, response surface methodology, activated sludge, bioaugmentation, adsorption, biodegradation

## Abstract

This study investigated the removal of SARS-CoV-2 antivirals substances (SASs) in wastewater treatment systems through kinetic analysis and response surface modeling based on a full factorial experimental design (3^3^). Quadratic models best described the removal of most SASs, revealing nonlinear interactions among experimental factors and identifying the SASs mixture ratio as the key determinant of removal efficiency. Based on their removal behavior, the investigated SASs were classified into three groups: reversible adsorption (FAV, REM), enhanced bioavailability (DCV, DRV) and irreversible adsorption (LOP, RIT). LOP and RIT exhibited the highest adsorption affinities and formed the initial layer on activated sludge, whereas the limited removal of DRV and FAV reduced the overall treatment efficiency. The increased bacterial Colony-Forming Unit (CFU) suggests enhanced microbial activity, which may have promoted extracellular polymeric substance (EPS) production, contributing to reduced toxicity toward *Aliivibrio fischeri*. Finally, integrating RSM-derived polynomial models with artificial intelligence offers a promising approach for predicting and optimizing the removal of SARS-CoV-2 antivirals in conventional wastewater treatment plants.

## 1. Introduction

Antiviral drugs (SASs) play a crucial role in the preventing and treating viral infections and have become increasingly important following the emergence of new viral diseases worldwide. Viruses are major pathogens that cause a variety of serious diseases in humans, animals and plants. Although there is still no cure for many viral infections, some SASs effectively treat hepatitis C and HIV, and several SASs are being tested in the search for a cure for severe acute respiratory syndrome coronavirus 2 (SARS-CoV-2) [1]. Despite advanced drug development tools and stringent quality control procedures, only a small number of SASs have been approved for clinical use, primarily because of significant side effects and the development of viral resistance. At the same time, environmental concentrations of some SASs are continuously increasing, raising concerns about their persistence, ecotoxicity and potential contribution to the development of resistance in the environment [2]. Because human and animal organisms do not fully metabolize SASs, substantial amounts are excreted in urine and feces and subsequently discharged into sewage systems. As a result, both parent compounds and their metabolites are frequently detected in wastewater treatment plant effluents, where they may pose ecological risks upon entering aquatic environments. In the context of the increasing prevalence of viral diseases worldwide, particularly following the emergence of SARS-CoV-2, there is an urgent need for a comprehensive assessment of the occurrence, fate, removal efficiency and environmental risks associated with SASs [3].

Conventional wastewater treatment processes generally exhibit limited efficiency in removing SASs, primarily because of their persistence and low biodegradability. Consequently, numerous alternative treatment approaches have been investigated, including ozonation, photolysis, photocatalysis, adsorption, activated sludge processes and membrane bioreactors. Although some of these methods achieve high removal efficiencies, their large-scale implementation remains challenging because of several limitations, such as high operating and capital costs, high energy demand, the generation of secondary pollution, and the formation of potentially harmful by-products that require additional treatment or disposal [4]. Among the available treatment technologies, biological wastewater treatment, which relies primarily on biodegradation, is considered one of the most environmentally friendly and cost-effective approaches for removing SASs and other organic pollutants. However, the biodegradability of SASs varies considerably depending on their chemical structure. For example, favipiravir (FAV) and remdesivir (REM) have been reported to exhibit high resistance to biodegradation, whereas lopinavir (LOP) and ritonavir (RIT) are more readily biodegradable [5,6]. Nevertheless, complete mineralization of SASs is rarely achieved. Instead, biodegradation often results in the formation of intermediates, some of which may be more toxic than their parent compounds [7,8]. Given the limited biodegradability of many SASs and the incomplete mineralization achieved by conventional biological treatment processes, strategies to enhance microbial degradation have attracted increasing attention. Among these, bioaugmentation has emerged as a promising approach for improving the biodegradation of persistent contaminants. It involves introducing specialized microbial strains capable of degrading target pollutants into contaminated environments or biological treatment systems [9]. By increasing the abundance and metabolic activity of functional microorganisms, this approach can improve the removal efficiency of recalcitrant compounds when the indigenous microbial community alone cannot achieve satisfactory degradation [10]. Several bacterial genera, including *Pseudomonas*, *Bacillus*, *Rhodococcus* and *Comamonas*, have demonstrated considerable potential in bioaugmentation processes. Their broad catabolic capabilities, remarkable metabolic versatility and ability to produce a diverse repertoire of degradative enzymes enable the biodegradation of a wide range of pharmaceuticals and other organic micropollutants, such as SASs [11,12]. Despite its numerous advantages, including relatively low operating costs, environmental sustainability, and the ability to enhance the biodegradation of target pollutants, bioaugmentation is often constrained by the limited survival, establishment, and long-term persistence of the introduced microorganisms. These limitations are primarily attributed to competition with indigenous microbial communities, predation, washout, and fluctuations in environmental and operational conditions [9,10]. The application of bioaugmentation has expanded from the degradation of individual pollutants to the treatment of complex mixtures of structurally diverse contaminants, including pharmaceuticals, where the combined metabolic capabilities of the microbial community play a key role in effective biodegradation. Recent studies suggest that successful bioaugmentation depends not only on the introduction of suitable microorganisms but also on optimizing the environmental and operational conditions that govern their survival, activity, and interactions with the indigenous microbial community. This perspective shifts the focus from treating bioaugmentation as a one-time inoculation procedure to deliberately shaping and optimizing microbial ecosystem functions over time [13]. Although the application of bioaugmentation in activated sludge systems for the removal of individual SASs and their mixtures remains insufficiently investigated, existing studies indicate that this approach can significantly enhance the biodegradation of other pharmaceutical compounds [14,15,16]. Consequently, the use of bioaugmentation to enhance the biodegradation of SAS mixtures represents a significant research gap. However, the efficiency of bioaugmentation in activated sludge systems cannot be evaluated independently of adsorption, as adsorption determines the partitioning and bioavailability of pharmaceuticals and thus directly influences their accessibility to microbial degradation.

Previous studies have investigated the adsorption of pharmaceuticals onto natural environmental matrices, sewage sludge and biological wastewater treatment systems [17,18,19]. However, information on the adsorption behavior of the SASs examined in the present study, particularly in activated sludge systems, remains limited. Sorption capacity strongly depends on the physicochemical properties of both the adsorbate and the adsorbent, as well as on process conditions. Proposed adsorption mechanisms include hydrophobic interactions, π–π interactions, π-electron donor–acceptor (π–EDA) interactions, van der Waals forces, ion exchange, surface complexation, electrostatic interactions, and hydrogen bonding [18]. These interactions govern the environmental fate of pharmaceuticals and should therefore be considered when evaluating the behavior and biodegradation of SASs in activated sludge systems [17,19]. Within the group of SASs investigated in this study, DRV, LOP, and RIT are classified as HIV-1 protease inhibitors, which inhibit the viral protease required for the maturation of infectious virus particles [20]. Although they belong to the same pharmacological class, differences in their molecular and physicochemical properties may result in distinct environmental behavior. Direct evidence of strong sorption to sewage sludge is available for LOP and RIT, which show high distribution coefficients (*K*_d_ = 2076–3449 L/kg), low desorption (<15%), and competition for sorption sites when present together [18], while anaerobic digestion of RIT leads to partial degradation, with 50.2–68.8% remaining in the digested sludge [21]. In contrast, comparable data on the sorption and biodegradation of DRV in sludge systems are still limited. Although adsorption contributes to the apparent removal of pharmaceuticals from the aqueous phase, it does not necessarily represent their complete elimination from the environment. Pharmaceuticals retained in activated sludge may persist, undergo only partial biodegradation, or be released back into the aquatic environment, thereby influencing their long-term environmental fate and potential ecological risks.

The environmental persistence of SASs, resulting from incomplete biodegradation and their retention on solid matrices, increases the likelihood of their long-term accumulation and eventual release into aquatic environments. Consequently, even low environmental concentrations may lead to prolonged exposure of non-target organisms, making the evaluation of their ecotoxicological effects particularly important. Studies have shown that the ecotoxicity of SASs primarily manifests through effects on aquatic organisms at various trophic levels, including algae, rotifers, crustaceans, and fish [3]. It is also important to recognize that SASs occur in the environment as mixtures rather than as individual compounds. Interactions among multiple SASs may result in additive, synergistic, or antagonistic effects, so risk assessments based solely on individual compounds may underestimate the actual risks associated with combined exposure [22,23]. Therefore, investigating mixtures of SASs is essential for realistically evaluating both their environmental fate and their combined ecotoxicological effects. In addition, because biodegradation may generate transformation products with altered or even increased toxicity, assessing post-treatment ecotoxicity is equally important for evaluating the environmental safety of the treatment process. Therefore, integrating biodegradation, adsorption, and post-treatment ecotoxicity assessment is essential for comprehensively evaluating both treatment efficiency and environmental safety.

The aim of this study was to optimize the removal of a mixture of SARS-CoV-2 antivirals, including DCV, DRV, FAV, LOP, REM and RIT, using bioaugmented activated sludge. The study further investigated the adsorption behavior of these antivirals onto activated sludge to elucidate their removal mechanisms and assessed the ecotoxicity of the treated effluent using *Aliivibrio fischeri*, thereby providing an integrated evaluation of treatment efficiency and environmental safety.

## 2. Materials and Methods

### 2.1. SAS Standards

All standards used in this research were high-purity solid analytical standards, stored at the recommended low temperatures. Stock solutions were prepared individually at concentrations of 2.00 μmol/L in the mineral medium described in Section 2.3. Final SAS concentrations for the different mixtures in the working solutions were obtained by diluting the stock solutions, as described in Section 2.5. The SAS standards included:

- DCV (98%, BLD Pharmatech Ltd., Shanghai, China);
- DRV (≥ 98%, Sigma-Aldrich, St. Louis, MO, USA);
- FAV (98%, Fluorochem Ltd., Hadfield, UK);
- LOP (≥ 98%, Sigma-Aldrich, St. Louis, MO, USA);
- REM (≥ 98%, Sigma-Aldrich, St. Louis, MO, USA);
- RIT (98%, Indagoo Research Chemicals, Madrid, Spain).

### 2.2. Activated Sludge

Raw activated sludge was collected from a municipal wastewater treatment plant in Zagreb, Croatia. Fresh sludge was characterized by determining its physicochemical properties, including pH, temperature (*T*), electrical conductivity (EC), dissolved oxygen (DO), water content, dry matter, volatile matter, mixed liquor suspended solids (MLSS) and mixed liquor volatile suspended solids (MLVSS), as described previously [24]. The initial bacterial abundance was determined by colony-forming unit (CFU) enumeration to establish the baseline microbial population prior to bioaugmentation. Before the optimization experiments, the activated sludge was washed three times with distilled water, allowing the biomass to settle after each washing step before removing the supernatant. The biomass was then concentrated by centrifugation (Universal 320/320R, Hettich^®^, Tuttlingen, Germany) at 3500 rpm and 0 °C for 10 min to obtain a higher initial MLSS concentration. The concentrated sludge was bioaugmented and acclimated for 24 h under aerobic conditions in a mineral medium containing a mixture of the investigated SASs at 25 ± 2 °C in a shaking incubator (Biobase BJPX-SDT20, Biobase, Jinan, Shandong, China) operating at 160 rpm. Throughout the acclimation period, pH and dissolved oxygen remained stable, indicating the absence of detectable microbial inhibition. Following acclimation, the sludge was washed three times with distilled water to remove residual antivirals. The concentrations of the investigated SASs in the final washing supernatant were determined using a Vanquish Flex UHPLC system (Thermo Fisher Scientific, Waltham, MA, USA) equipped with a diode array detector (DAD FG, VF-D11-A-01). The analysis confirmed the complete removal of residual antivirals before the bioaugmented sludge was used in the biodegradation experiments.

### 2.3. Mineral Medium

The mineral medium used to provide the necessary nutrients to the microorganisms during the experiments was prepared according to Kyaw et al. [25], as in our previous studies, to ensure comparable data [24]. The composition of the mineral medium is given in Table 1.

The phosphate buffer composition was determined using the Henderson–Hasselbalch equation, which is commonly used for buffer pH calculations [26]. Although the experiments were conducted at pH 6, which is at the lower limit of the buffer’s working range, pH control should remain well balanced. When necessary, the pH was adjusted with 1 mol/L HCl or 1 mol/L KOH.

### 2.4. Inoculum for Bioaugmentation

Pure cultures of *Comamonas testosteroni* (B1), *Bacillus amyloliquefaciens* (B2) and *Bacillus mycoides* (B3), previously isolated and characterized by Lastovčić et al. [27], were used for activated sludge bioaugmentation. For simplicity, the corresponding bioaugmentation treatments were designated as B1, B2, and B3 according to the inoculated bacterial strain. Prior to the experiments, each strain was cultivated on nutrient agar at 37 °C for 24 h and resuspended in the mineral medium described above to obtain highly concentrated bacterial inoculum. The inoculum was subsequently diluted to the optical densities (OD600) specified by the full factorial design (FFD) (Section 2.6) and mixed with the concentrated activated sludge (Section 2.2). Following inoculation, the bioaugmented activated sludge was acclimated to a mixture of the investigated SASs as described in Section 2.2 before being used in the optimization experiments. The OD600 of the prepared inoculum was determined using a portable spectrophotometer (DR/2400, Hach, Loveland, CO, USA) and presented in Table A2.

### 2.5. Experimental Setup

**Figure 1:**
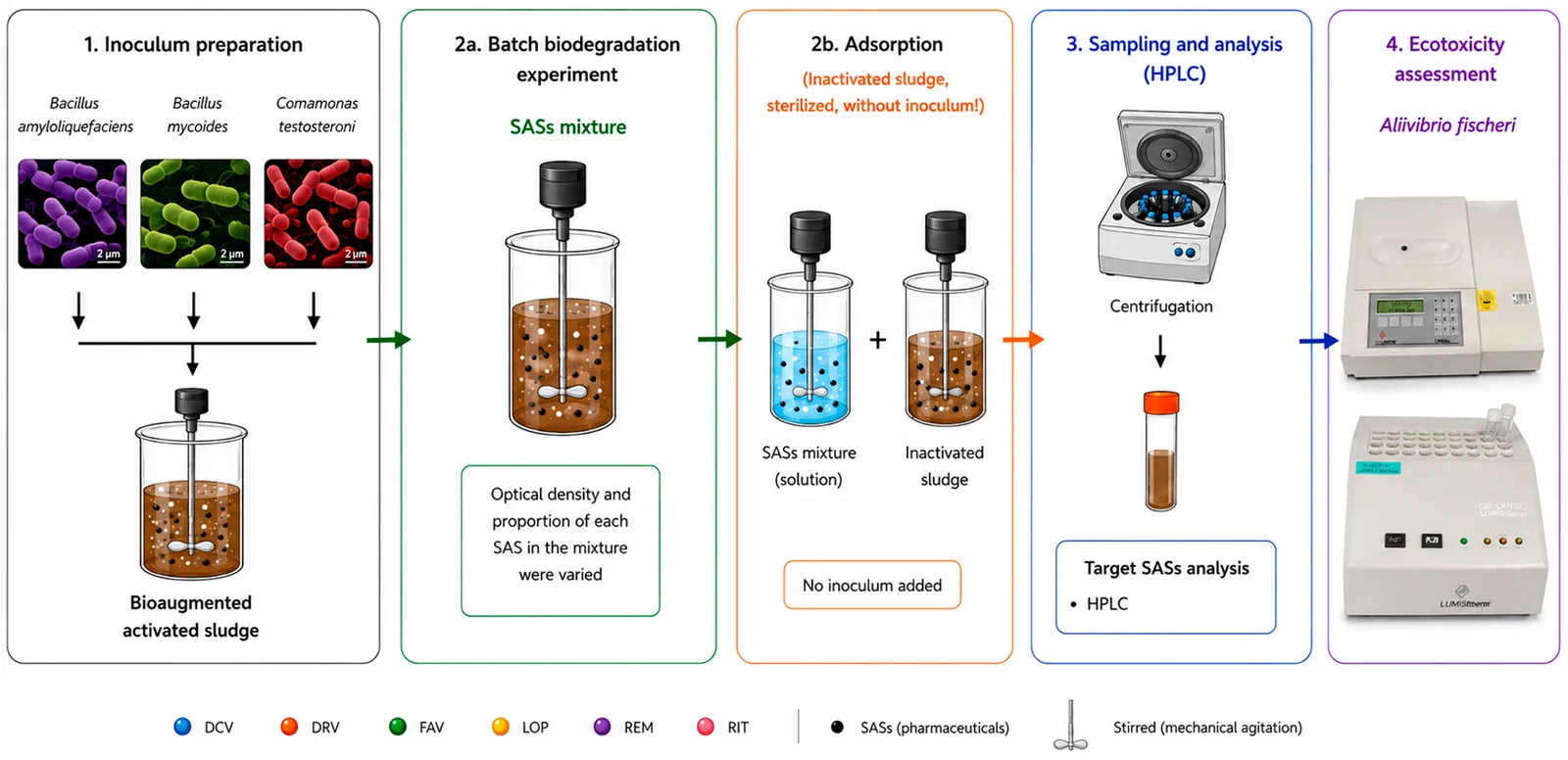
Schematic diagram illustrating the experimental preparation and workflow.

The optimal operating conditions for the biodegradation of the investigated SASs (pH 6, MLSS 4 g/L, and 45 °C) were adopted from our previous study [24] and applied for the optimization of the bioaugmentation process. Bioaugmentation experiments were performed in triplicate in 250 mL Erlenmeyer flasks containing 150 mL of the reaction mixture. Each flask contained acclimatized bioaugmented activated sludge, mineral medium, and one of three SAS mixtures (Figure 1). The amount of centrifuged activated sludge (8.89 g) was adjusted according to the procedure described by Lastovčić et al. [24] to achieve the target MLSS concentration of 4 g/L. Control experiments were also performed in triplicate under identical conditions without the addition of the SAS mixtures to verify the stability of the bioaugmented activated sludge. All flasks were incubated in a shaking incubator (Biobase BJPX-SDT20, Biobase, Jinan, Shandong China) at 45 °C and 160 rpm for 48 h. The composition of each mixture was prepared according to Table 2, while the total SAS concentration was maintained at 6.00 μmol/L in all experiments. Mixture M1 represented a symmetrical mixture containing all six SASs at equal concentrations (1:1 ratio). Mixture M2 was designed as a DRV-enriched mixture based on our previous findings, which identified DRV as the rate-limiting compound during biodegradation by activated sludge [24]. Accordingly, DRV accounted for 50% of the total SAS concentration, while the remaining five antivirals shared the other 50%. Mixture M3 was designed as a protease inhibitor-enriched mixture to evaluate the potential inhibitory effects of structurally related compounds. In this mixture, DRV, LOP and RIT each represented 25% of the total SAS concentration, whereas DCV, FAV and REM shared the remaining 25%.

The biodegradation process was monitored by determining the concentrations of the investigated SASs and measuring DO, pH, EC, MLSS and MLVSS. SAS concentrations were quantified using the UHPLC-DAD method described in Section 2.8. Dissolved oxygen, pH, and EC were measured using electrodes connected to a portable multiparameter meter (WTW Multi 340i, WTW, Weilheim, Germany). Activated sludge samples were microscopically examined after 6, 24, and 48 h using a light microscope (Olympus B201, Tokyo, Japan), while MLSS and MLVSS were determined according to the standard methods described by Lastovčić et al. [24].

The samples were collected at 0, 1, 2, 4, 6, 8, 12, 24, 30, and 48 h and prepared for HPLC analysis by centrifugation at 2800 rpm for 5 min (FV-2400 Minicentrifuge-Vortex Microspin, SIA Biosan, Riga, Latvia) to separate the suspended solids. The resulting supernatant was filtered through a new pre-wetted 0.45 μm PTFE membrane filter for each sample to prevent SAS retention on the membrane and cross-contamination between samples.

### 2.6. Full Factorial Design (3^3^)

The bioaugmentation process was optimized using a full factorial design (FFD) generated in Design-Expert^®^ 12 software (Stat-Ease Inc., Minneapolis, MN, USA) as presented in Table A1 and summarized in Table 3. The experimental design included three factors, one numerical (optical density, OD600) and two categorical (SAS mixture and bacterial inoculum), each evaluated at three levels. The numerical factor (OD600) was tested at 0.1, 0.5, and 1.0, whereas the categorical factors comprised three SAS mixtures (M1, M2, and M3) and three bacterial strains (B1, B2, and B3). The removal efficiencies of the individual SARS-CoV-2 antivirals were selected as the response variables.

RSM was applied to the experimental data (T1–T27, Table A1) to evaluate the effects and interactions of the investigated factors on the removal of individual SASs and to determine the optimal bioaugmentation conditions. Three-dimensional response surface plots and statistical analyses were generated using Design-Expert^®^ 12 software (Stat-Ease Inc., Minneapolis, MN, USA). For each response, the initial model type was selected according to the Fit Summary provided by the software. The selected models were subsequently evaluated and refined based on analysis of variance (ANOVA), goodness-of-fit statistics (*R*^2^, adjusted *R*^2^, predicted R^2^), adequate precision and lack-of-fit. Where appropriate, statistically identified outliers based on externally studentized residuals were excluded, and non-significant interaction or polynomial terms (*p* > 0.05) were removed to improve model performance while preserving the biological relevance of the experimental data. Because the experimental design comprised 27 runs, no more than three experimental points were excluded during model refinement. Only statistically significant models were used to interpret factor interactions and identify the optimal bioaugmentation conditions.

### 2.7. Adsorption Test

Adsorption experiments were performed using sterilized activated sludge to eliminate the contribution of biodegradation. The amount of adsorbent corresponded to the dry matter content of the activated sludge determined during sludge characterization (Section 2.2). The experiments were carried out as abiotic controls under the same operating conditions as the bioaugmentation experiments (45 °C, 160 rpm, 48 h). Sterilized activated sludge was suspended in mineral medium containing the investigated SASs and samples were collected after 0, 1, 2, 4, 6, 8, 12, 24, 30, and 48 h. SAS concentrations were determined using the UHPLC-DAD method described in Section 2.8. The adsorption efficiency (*η*, %) was calculated from the initial (*c*_0_, μmol L^−1^) and final (*c*_48_h, μmol L^−1^) concentrations according to Equation (1):

(1) $$\eta =\frac{c_{0}-c_{48h}}{c_{0}}\times 100$$

### 2.8. HPLC Method

**Figure A1:**
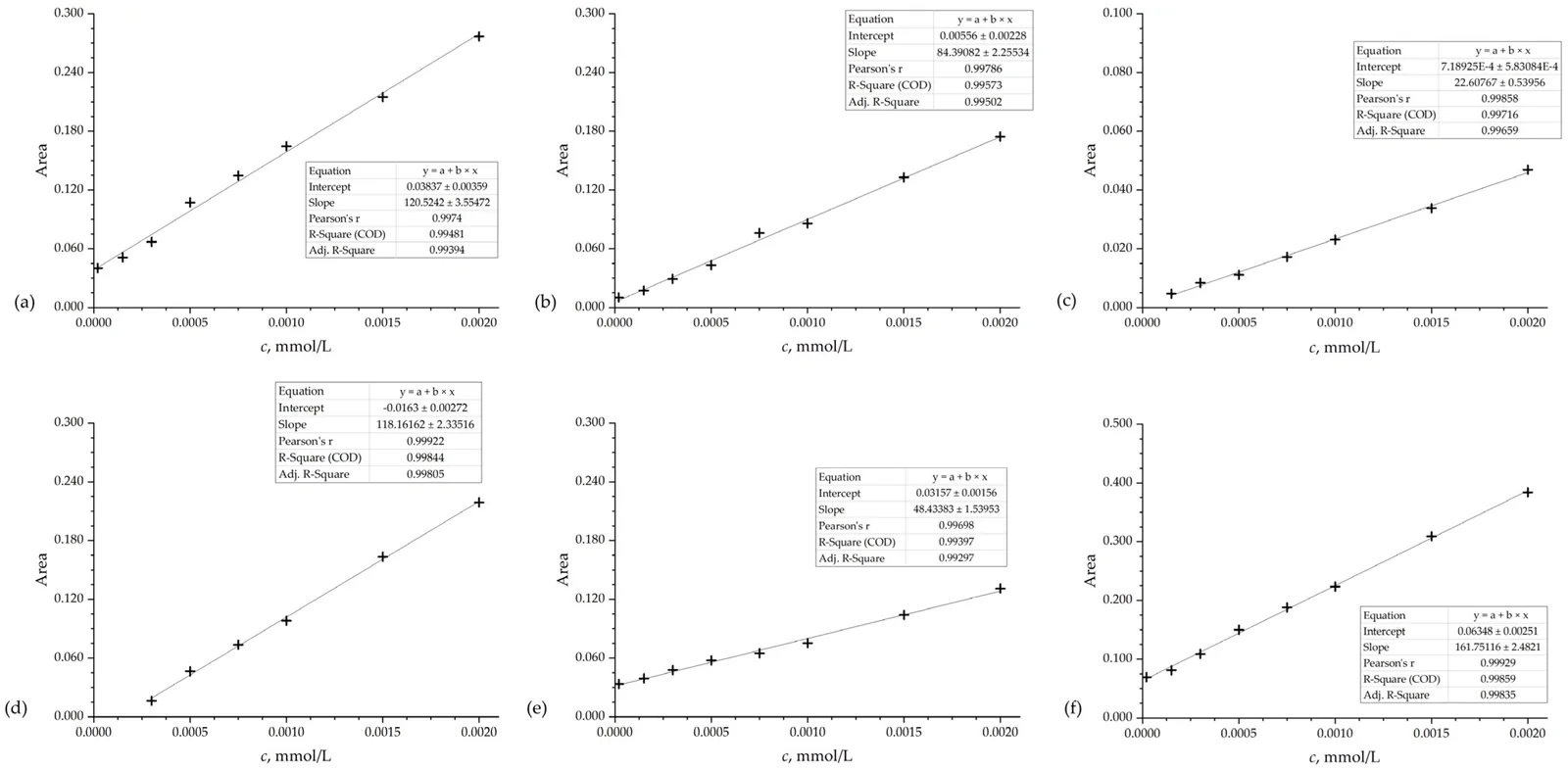
HPLC calibration curves for the six analyzed SASs used for quantification: (**a**) DCV; (**b**) DRV; (**c**) FAV; (**d**) LOP; (**e**) REM; (**f**) RIT;.

To detect each individual SAS in a mixed solution, a gradient HPLC method was developed using Chromeleon 7 software on the Vanquish Flex UHPLC system (Thermo Fisher Scientific, Waltham, Massachusetts, USA) equipped with a diode array detector (DAD FG, VF-D11-A-01). SAS standards were dissolved in HPLC-grade methanol with appropriate dilutions for individual calibration curves (Figure A1). The stationary phase was an XBridge C18 3.5 μm (2.1 × 150 mm) column, while the mobile phase was Mili-Q water with 0.1% formic acid (phase A) and acetonitrile (phase B). The method gradient was controlled by %B over time and is shown in Table 4, along with operational parameters such as flow rate, injection volume, and column temperature. Additional parameters and statistically relevant information for individual SASs are presented in Table 5.

### 2.9. Aliivibrio fischeri Toxicity Test

The inhibition test on the bioluminescence of *Aliivibrio fischeri* was conducted according to the standard protocol ISO 11348-1:2007 [28], titled Water Quality-Determination of the inhibitory effect of water samples on the light emission of *Aliivibrio fischeri* (luminescent bacteria test). Detailed information on the procedure can be found in our previous studies [24,27]. Bioluminescence was recorded with a LUMIStox 300 luminometer (Dr. Lange GmbH, Hannover, Germany) equipped with a LUMIStherm thermostat to maintain the incubation temperature at 15 °C. Samples were prepared by membrane filtration (0.45 μm, PTFE) on an already conditioned filter with Mili-Q water for each sample. The pH was adjusted to 7.0 ± 0.2 with 1 mol/L HCl or NaOH to eliminate pH-related inhibitory effects.

Inhibition was measured on 48 h sample filtrates. The results were presented as total inhibition percentages (*INH*, %) or, when possible, as concentrations causing 20% (*EC*_20_) and 50% (*EC*_50_) inhibition. *EC*_20_ and *EC*_50_ were determined by linearization of the dose-response curve (ln *γ*, ln *c* (SAS)), which included calculating the factor *γ* (%) according to Equation 2, and the logarithmic value of the linear dilution concentrations of each SAS.

(2) $$\gamma =\frac{INH}{\left(100-INH\right)}$$

### 2.10. Data Processing

All bioaugmentation and adsorption experiments were conducted and reported in triplicate. Results are presented as mean ± standard deviation (SD). Graphical visualization was performed using OriginPro 8.5 software (OriginLab Corporation, Northampton, MA, USA) and Microsoft Office Visio 2007. FFD and RSM were estimated and analyzed using the Design-Expert 12 software package.

## 3. Results

### 3.1. Characterization of Activated Sludge Prior to Bioaugmentation

**Figure 2:**
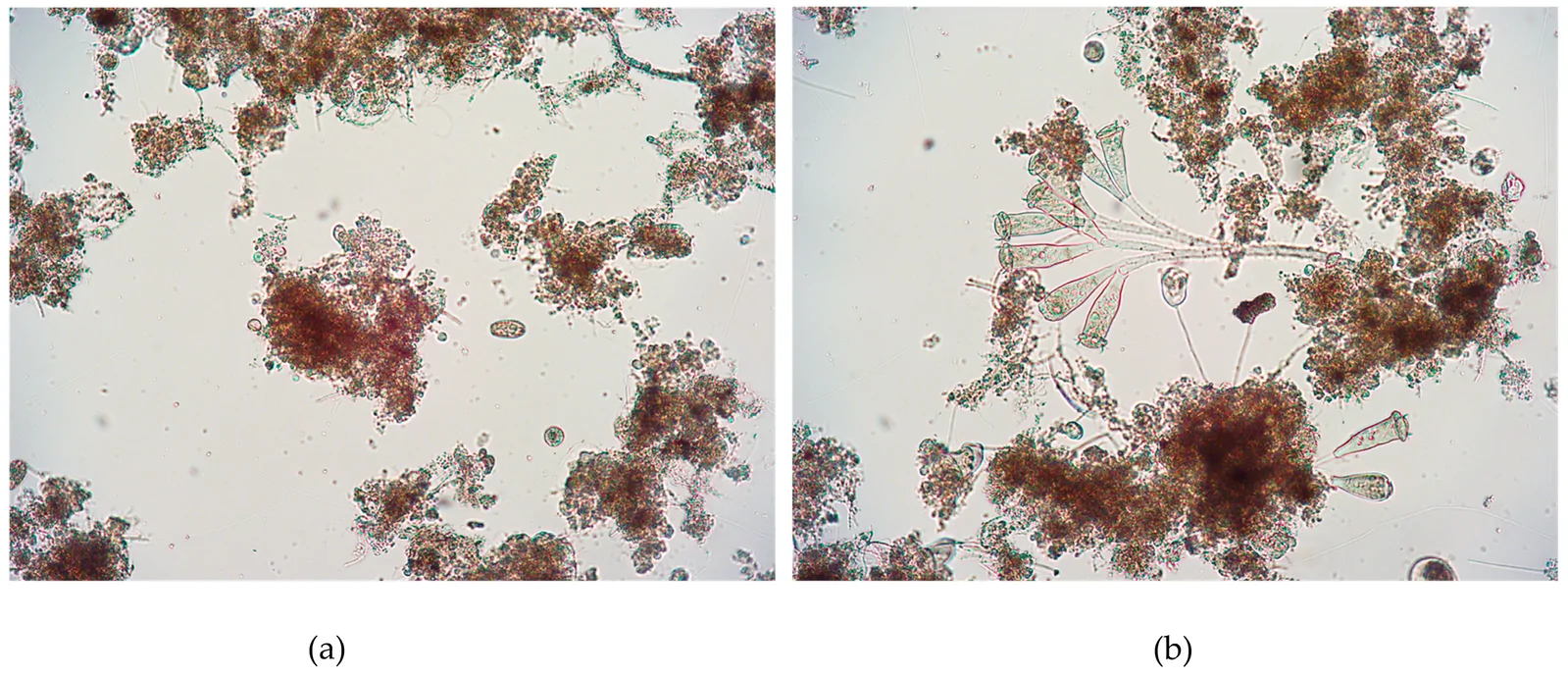
Microscopic observation of the activated sludge flocs: (**a**) general morphology and structural form of the flocs; (**b**) presence of ciliated protozoa (ciliates) attached to the floc structure.

Prior to bioaugmentation, the raw activated sludge was characterized to establish its initial physicochemical and microbiological properties. The key parameters are summarized in Table 6. The measured pH, *T*, DO and EC were within the ranges expected for activated sludge obtained from an operating wastewater treatment plant (WWTP) in Zagreb. Water content, dry matter, volatile matter, MLSS, MLVSS, and bacterial abundance expressed as CFU provided the baseline characteristics relevant to the subsequent bioaugmentation and adsorption experiments. The sludge contained more than 99% water, consistent with the highly hydrated structure of activated sludge flocs (Figure 2).

Consistent with the high-water content (>99%), the dry matter fraction was below 1%, which was taken into account when determining the adsorbent mass used in the adsorption experiments. The high volatile matter content and MLVSS values further indicated that the activated sludge was predominantly composed of organic biomass, with a relatively low contribution of inorganic matter. The initial MLSS concentration of the raw WWTP sludge was 3.50 ± 0.58 g/L, which was close to the target concentration of 4 g/L previously established as optimal for the bioaugmentation experiments [24].

In addition to these physicochemical characteristics, the abundance of culturable bacteria provided an important microbiological baseline for evaluating the subsequent bioaugmentation process. The raw activated sludge contained approximately 10^6^ bacterial CFU/mL, as determined at a 10^−5^ dilution, indicating an abundant indigenous bacterial population. Bacteria represent a major functional component of activated sludge microbial communities and play a key role in the biodegradation of organic contaminants through their diverse metabolic pathways and enzymatic activities. Their abundance and metabolic diversity are therefore particularly relevant to the transformation and removal of pharmaceuticals, including SASs. The substantial indigenous bacterial population observed in the raw sludge also provided a relevant background against which the contribution of the introduced bacterial strains B1, B2, and B3 could be evaluated. These strains, previously isolated using SASs as the sole carbon source, were selected for their potential to complement the degradative capacity of the indigenous activated sludge community. The presence of the introduced bacterial cultures was monitored daily in relation to the initial bacterial consortium. The inoculum levels defined by the experimental design corresponded to OD600 values of 0.1, 0.5, and 1.0, representing approximately 10^6^–10^7^, 10^7^–10^8^, and 10^8^ cells/mL, respectively.

### 3.2. Bioaugmentation Operational Parameters

#### 3.2.1. pH

**Figure 3:**
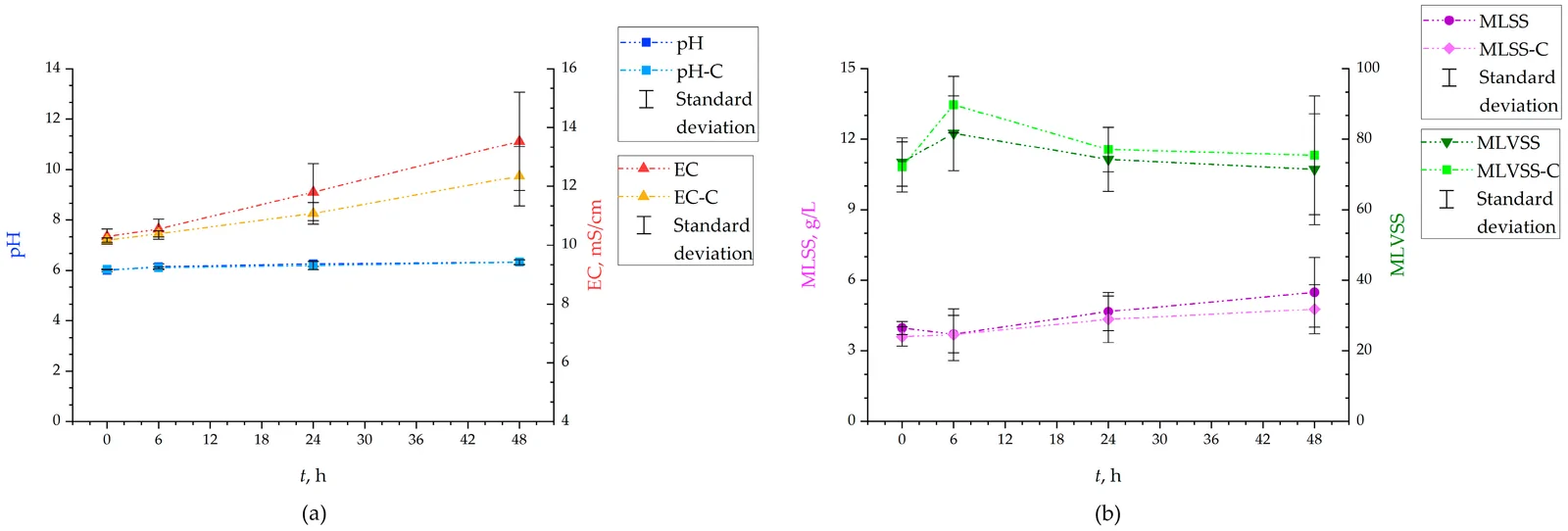
Operational parameter profiles during the experiments: (**a**) monitoring of pH and EC; (**b**) dynamic variations of MLSS and MLVSS.

The experiments were conducted at an optimal pH of 6, maintained by adjusting the phosphate buffer included in the mineral medium (Section 2.3). Figure 3a and Table A3 present the mean pH values for all triplicates, which averaged 6.18 ± 0.15 across all T1–T27 samples. More specifically, pH remained stable over 48 h, as the tested solutions and controls followed an increasing trend with a difference below 0.50 units (0 h: 6.00 ± 0.05; 48 h: 6.33 ± 0.09). However, a few exceptions were noted when comparing mean pH values of individual tests. T3 (1, 0, −1) showed the lowest pH value (6.07 ± 0.13), while T25 (1, −1, 1) had the highest value (6.34 ± 0.18) during the 48 h of testing, suggesting greater oscillation in pH levels with higher DO values (especially 1.0). These observations are also reflected in the mean values for the individual B1, B2, and B3 experiments. Compared to the controls (6.16 ± 0.14), B1 and B2 showed similar pH of 6.17 ± 0.15, while B3 had a pH of 6.21 ± 0.16, which was only slightly higher than the controls. Examining the controls of uninoculated sludge with M1, M2, and M3, the control with M1 showed an increase in pH up to 24 h (from 6.05 ± 0.04 to 6.38 ± 0.01), followed by a slight decrease at 48 h (6.21 ± 0.06). Although this pattern was not observed in T1–T27, closer inspection and comparison between B1, B2, and B3 showed that bioaugmentation tests with M2 and M3 had lower pH values (6.11 ± 0.07), particularly with the OD combination of 0.5 to 1.0 (T3–T4, T12–T13, T21–T22), while a slightly higher pH (6.25 ± 0.16) was measured for T6, T15, and T24, which were conducted with M1. Overall, the small pH variations observed among the different bacterial inoculum, OD levels, and SAS mixtures indicate effective buffering of the system, while the slightly lower pH values observed for M2 and M3 may reflect differences in microbial metabolic activity associated with the composition of these mixtures.

#### 3.2.2. DO

Table A4 summarizes the changes in DO throughout the bioaugmentation experiments. The largest increase was observed in T21 (1, 0, 1), where DO increase by 3.25 mg/L relative to the initial value. Similar increases of 2.00–3.16 mg/L were recorded for T2, T10–T12, T18, and T20, predominantly associated with the M2 and M3 mixtures. In contrast, the uninoculated activated sludge controls maintained relatively stable DO concentrations, with variations within ±0.67 mg/L throughout the experimental period, indicating limited changes in oxygen consumption in the absence of bioaugmentation. A different DO profile was observed for T6–T8, T15–T17 and T23–T26, where DO concentrations decreased during the experiments. These decreases were predominantly associated with the symmetrical M1 mixture across the tested bacterial strains and may indicate increased microbial oxygen consumption resulting from enhanced metabolic activity. Such oxygen depletion could be associated with aerobic transformation of the SASs. Of particular interest was T23, representing the B3–M3 combination, which showed a decrease in DO and may indicate a higher metabolic response of B3 toward the protease inhibitor-enriched mixture compared with the other bacterial strains. Overall, the mean DO concentration across the bioaugmentation experiments (T1–T27) was 0.67 mg/L lower than that of the controls (C1–C3). The consistently lower DO levels in the bioaugmented systems suggest greater oxygen consumption and consequently, increased aerobic microbial activity following bacterial inoculation.

#### 3.2.3. EC

The EC profiles of the bioaugmentation experiments (T1–T27) and controls (C1–C3) are presented in Figure 3a and Table A5. The initial EC was relatively uniform across all experiments (10.29 ± 0.25 mS/cm) and gradually increased to 13.41 ± 1.64 mS/cm after 48 h. The increasing SD over time indicates that differences among the experimental conditions became more pronounced as the bioaugmentation process progressed. In contrast, the controls exhibited comparatively smaller changes, suggesting that the observed EC variations were influenced by the composition and activity of the bioaugmented systems. Compared to the controls, T6 (0, −1, −1) exhibited the most pronounced deviation, with a marked decrease in EC after 24 h followed by a subsequent increase toward the initial value. Nevertheless, its final EC remained below that of the corresponding controls. Approximately half of the experiments showed EC profiles comparable to those of the controls, whereas a gradual increase was observed in T1–T2 (B1), T15–T16 (B2), and T19 and T22–T26 (B3). For B1, increased EC was primarily observed at lower inoculum levels (OD600 = 0.1–0.5) in the presence of M2 and M3, whereas the M1-containing system T6 showed a decrease in both EC and DO. This pattern may reflect greater microbial utilization of ionic nutrients from the mineral medium under these conditions, although EC changes alone cannot distinguish between nutrient uptake and other biological or physicochemical processes. In contrast, increased EC in the B2 experiments was mainly associated with higher inoculum levels (OD600 = 0.5–1.0) and the M1 mixture, whereas B3 showed increasing EC for both M1 and M3 across different inoculum levels. Overall, the mean EC increased progressively from B1 (11.25 ± 1.44 mS/cm) and B2 (11.47 ± 1.43 mS/cm) to B3 (11.91 ± 1.90 mS/cm), while the controls showed a lower mean value (11.00 ± 1.00 mS/cm). The higher EC observed in the bioaugmented systems may reflect an increased release of ionic species associated with microbial metabolism, transformation of organic matter or biomass turnover.

#### 3.2.4. MLSS

The MLSS profiles of the bioaugmented and uninoculated activated sludge are presented in Figure 3b and Table A6. During the first 6 h, MLSS decreased across both the bioaugmentation experiments and controls, from an initial mean value of 3.94 ± 0.30 g/L to 3.71 ± 0.81 g/L. This initial decrease may reflect a short adaptation and stabilization phase following transfer to the experimental conditions, potentially involving biomass restructuring and partial loss or transformation of suspended solids. Subsequently, MLSS gradually increased, reaching an overall mean of 5.41 ± 1.45 g/L after 48 h. The most pronounced increases at 48 h were observed for T16 (1, −1, 0), T23 (1, 1, −1) and T25 (1, −1, 1), with MLSS values of 8.06 ± 0.57, 8.46 ± 0.75, and 9.64 ± 0.78 g/L, respectively. These results indicate a greater accumulation of suspended solids in systems bioaugmented with B2 and, particularly, B3 compared with B1. Overall, the bioaugmented systems exhibited higher MLSS values than the uninoculated controls, which had a mean concentration of 4.10 ± 0.94 g/L. Mean MLSS increased from 4.20 ± 0.86 g/L for B1 to 4.44 ± 0.96 g/L for B2 and 4.74 ± 1.51 g/L for B3, indicating a strain-dependent effect on solids accumulation during bioaugmentation.

#### 3.2.5. MLVSS

To further distinguish changes in the organic biomass fraction from the overall accumulation of suspended solids reflected by MLSS, MLVSS values were evaluated and are presented in Figure 3b and Table A7. Among the monitored operational parameters, MLVSS exhibited the greatest variability across the experimental conditions. Initial values were relatively uniform (73.36 ± 6.79%), whereas pronounced differences emerged during the 48 h experiments in both the controls and bioaugmented systems (T1–T27), indicating substantial changes in the organic fraction of the activated sludge. The controls showed mixture-dependent MLVSS profiles. The M1 and M2 controls initially increased to 97.18% and 91.91%, respectively, at 6 h, followed by decreases to 71.43% and 60.89% after 48 h. In contrast, the M3 control showed a gradual increase from 80.00% to 93.87%. These differences indicate that the composition of the SAS mixture itself influenced the organic fraction of the uninoculated activated sludge, even in the absence of bioaugmentation. However, changes in MLVSS alone cannot be directly attributed to bacterial growth, as they may also reflect biomass turnover, changes in floc composition, or degradation and accumulation of organic suspended matter.

Considering the changes between the initial and final MLVSS values relative to the corresponding controls, T9 showed the greatest decrease, whereas T15 exhibited the most pronounced increase within the full factorial design. For M1, the highest MLVSS values were generally associated with an intermediate inoculum level (OD600 = 0.5), with average increases of 16.14%, 17.97%, and 13.08% for B1, B2, and B3, respectively, compared with the control. In contrast, lower MLVSS values were observed for T8, T17, and T25 at OD600 = 0.1, suggesting that the lowest inoculum level may have been insufficient to maintain or increase the organic biomass fraction under these conditions. The M2 experiments showed predominantly negative changes in MLVSS, ranging from a 17.12% decrease in T20 to a 1.18% increase in T3. The most pronounced reduction was observed for T9, with MLVSS decreasing by 48.35% relative to the corresponding control. Considering that DRV accounted for 50% of the total SAS concentration in M2, this response suggests that the combination of the DRV-enriched mixture and the lowest B1 inoculum level (OD600 = 0.1) was unfavorable for maintaining the organic biomass fraction. In contrast, M3 produced the most consistent MLVSS response, with the experimental values generally following the trend observed for the corresponding control and reaching 59.46 ± 6.88% after 48 h.

### 3.3. Comparison of Removal Kinetics and Adsorption Effects

**Figure 4:**
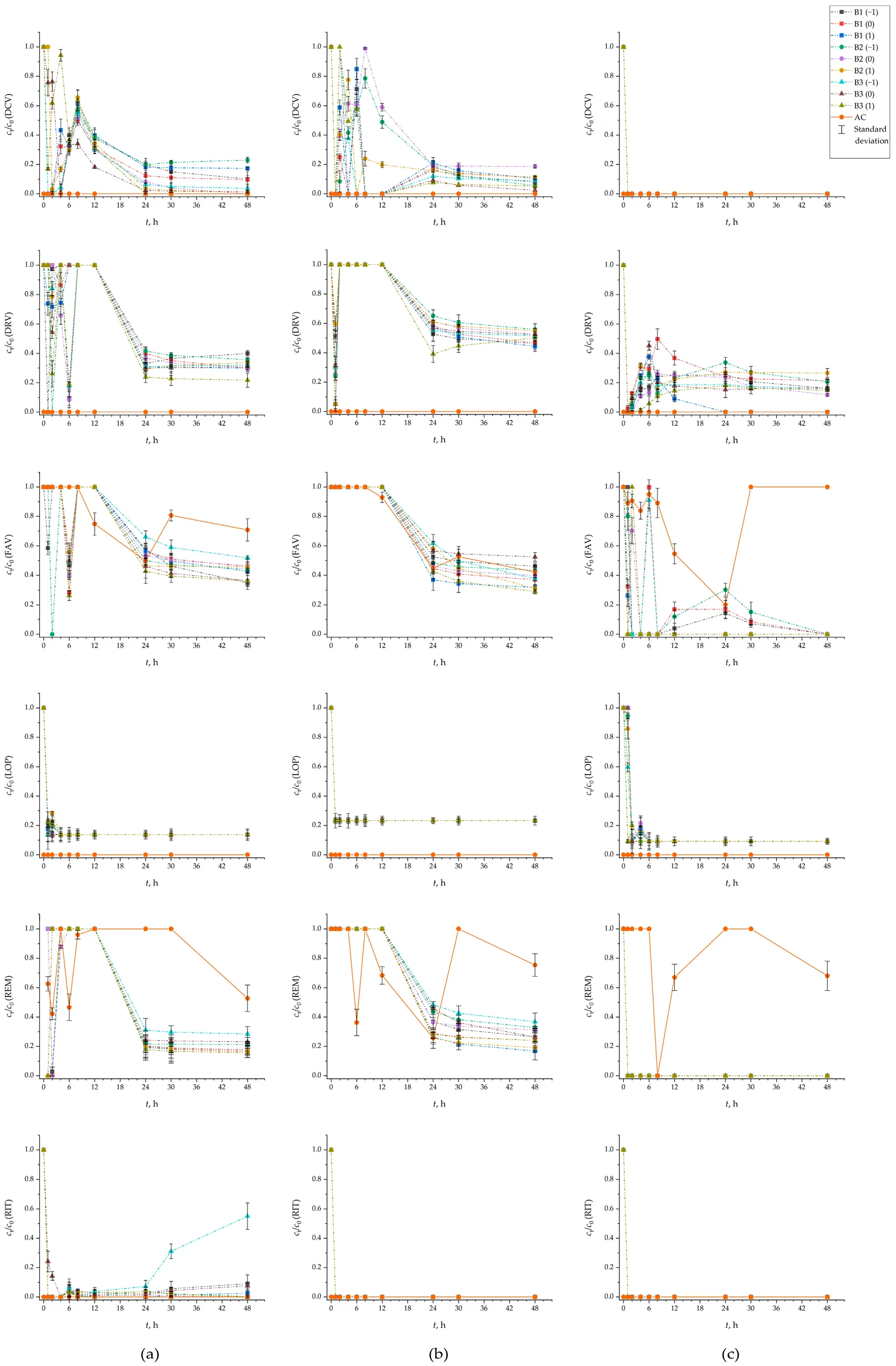
Removal kinetics of SASs in different configurations: (**a**) mixture M1 kinetics; (**b**) mixture M2 kinetics; (**c**) mixture M3 kinetics.

The removal profiles of individual SASs from the multicomponent mixtures, together with their adsorption kinetics, are presented in Figure 4. The observed temporal profiles indicate distinct kinetic phases during SAS removal. An initial period of slower removal was observed during the first 6–12 h, depending on the experimental conditions and the individual SAS, which may be associated with microbial adaptation to the experimental conditions and the presence of the investigated compounds. This was followed by a period of more rapid SAS removal (log phase). However, considering the contribution of adsorption demonstrated in the corresponding controls, these concentration profiles should be interpreted as apparent removal kinetics rather than exclusively as biodegradation kinetics.

For clarity, the results are grouped according to mixture composition as M1 (Figure 4a), M2 (Figure 4b), and M3 (Figure 4c), allowing direct comparison of bioaugmentation and adsorption profiles for each SAS. Distinct removal patterns were observed among the investigated compounds, indicating differences in their behavior during bioaugmentation and adsorption.

While our previous study [24] focused on the biodegradation of individual SASs, the present results demonstrate that their behavior changes when they are present in multicomponent mixtures. This effect was particularly evident in the adsorption profiles, suggesting that interactions among coexisting SASs influence their affinity for activated sludge and consequently their overall removal behavior. Distinct adsorption and removal patterns were observed among the investigated SASs. While DRV and RIT showed nearly complete removal in the abiotic controls, indicating a strong affinity for activated sludge, FAV and REM exhibited pronounced fluctuations in aqueous-phase concentrations over time. These fluctuations suggest reversible sorption–desorption behavior, potentially driven by redistribution among available sorption sites and competitive interactions between SASs in the multicomponent systems. Such competition may alter the accessibility and affinity of individual compounds for binding sites on the activated sludge. RIT exhibited particularly strong removal behavior, as it was almost completely removed from all three mixtures (M1–M3) in both the bioaugmentation and adsorption experiments. Its rapid disappearance from the aqueous phase suggests that sorption onto activated sludge was a major initial removal mechanism. A similar rapid initial removal was observed for most SASs in M3. In contrast, FAV and DRV showed high removal during the first 1–6 h of the bioaugmentation experiments (T1–T27), followed by an increase in their aqueous-phase concentrations and stabilization or reduction of removal efficiency toward 48 h. This behavior may indicate initial sorption followed by partial desorption or redistribution as competition for sorption sites developed within the multicomponent system.

In contrast to M3, M1 and M2 exhibited generally similar kinetic profiles, which may be attributed to their comparable composition, with the main difference being the higher proportion of DRV in M2. Particularly interesting behavior was observed for DCV and DRV. Based on the abiotic adsorption experiments, both compounds were expected to be strongly retained by the activated sludge; however, distinct concentration profiles remained observable in the bioaugmented systems. Their greater presence in the aqueous phase compared with the abiotic controls suggests that bacterial inoculation altered their partitioning between the sludge and aqueous phases, potentially increasing their bioavailability for subsequent microbial transformation. This effect may be associated with changes in the sludge microenvironment induced by microbial activity, including the production of EPS and extracellular metabolites or enzymes, although the contribution of these mechanisms was not directly quantified. For the remaining SASs, minor differences between the bioaugmentation and adsorption profiles were observed, while their overall kinetic behavior remained comparable. Following the initial adaptation period, a general decrease in SAS concentrations was observed across all mixtures and bioaugmentation treatments (B1, B2, and B3). An exception was RIT in the M1–B3 system at the lowest inoculum level (OD600 = 0.1), where an increase in aqueous-phase concentration was observed after 24 h compared with experiments conducted at higher inoculum levels. This transient increase may reflect redistribution or partial desorption of RIT during the early adaptation of the bioaugmented system rather than insufficient inoculum alone. LOP exhibited a distinct removal profile across all three mixtures, characterized by rapid initial removal followed by only minor fluctuations during the first 1–6 h. Although LOP was almost completely removed in the abiotic controls, its concentration profiles in the bioaugmentation experiments generally paralleled those observed during adsorption, with removal subsequently stabilizing at approximately 80–90%. This similarity suggests that sorption represented a major removal mechanism for LOP, with a comparatively smaller contribution from biological transformation.

Based on the combined bioaugmentation and abiotic adsorption profiles, the investigated SASs were classified into three groups according to their characteristic removal behavior: (group 1) reversible sorption, represented by FAV and REM; (group 2) enhanced aqueous-phase bioavailability in the presence of bioaugmented sludge, represented by DCV and DRV; and (group 3) irreversible sorption, represented by LOP and RIT. This classification provides a framework for distinguishing the relative contributions of sorption, desorption, and biologically mediated processes to the overall removal of individual SASs from multicomponent systems.

#### 3.3.1. Group 1

FAV and REM were the only investigated SASs that exhibited dynamic trends in abiotic controls. Both FAV and REM showed relatively stable concentrations in solution with no adsorption in the first 6 h, and the first signs of removal appeared after 6–12 h in all mixtures. The only exception was REM in M1, which exhibited immediate and highly dynamic oscillations in adsorption during the first few hours. FAV also showed notable behavior in the M2 solution, as its adsorption trend closely followed the total removal of this SAS. Although there was a significant correlation, under some conditions FAV was detected at lower concentrations than in abiotic controls; therefore, biodegradation was still present, but only with certain bacteria and at specific OD levels. Otherwise, the observed oscillations indicate that other SASs exhibited stronger adsorption to the activated sludge, with stronger binding than FAV and REM, strongly suggesting competitive adsorption and weaker bonds to the sludge for these two SASs than for others, especially because different kinetic trends were observed in different SAS mixture ratios. Although higher concentrations were observed in T1–T27 than in abiotic controls, this is linked to testing a live and active biological system compared to inactivated sludge, which was used only to discuss the binding potential of each SAS.

In the M3 solution, both FAV and REM showed substantial desorption at 48 h, with significant removal supporting biodegradation by the active microbial consortium. M1 proved to be the least effective option for removal of REM, while M2 was the least effective for FAV, as these experiments showed smaller final differences between adsorbed and removed concentrations.

#### 3.3.2. Group 2

DCV and DRV are considered to have distinct removal mechanisms; DCV is thought to be highly adsorbed onto the sludge, while DRV is associated with active biodegradation. Nonetheless, in our experiments with mixtures, they showed similar trends. In abiotic controls, both SASs were instantly removed from solution and bound to the sludge flocs. However, in the biologically active system, we observed overall exponential removal trends and unexpectedly high bioavailability in the first 6–12 h, with oscillations attributable to microorganism stabilization in response to the introduced toxic SASs. This suggests that different kinetic trends are directly related to the SAS ratios in mixtures, as DCV exhibited no bioavailability in M3, which contained higher concentrations of DRV, LOP, and RIT. In this mixture, it is also notable that higher desorbed and available concentrations of FAV and REM were observed in abiotic controls, while DCV in M3 experiments showed lower concentrations, possibly related to competitive adsorption between SASs on the sludge surface. DRV also exhibited lower bioavailability in this mixture than in M1 and M2 in the first 6 h; however, after 48 h, similar or lower removal was achieved as in M1 experiments.

For DCV, similar trends were observed in M1 and M2. However, for DRV we observed lower removal in M2 than in M1, which is related to the higher concentration of DRV in M2 (50% of the total mixture) compared to M1 (16.67% of the total mixture), as expected.

#### 3.3.3. Group 3

LOP and RIT were linked to irreversible adsorption, as all tests (T1–T27) exhibited immediate removal of these SASs in both biologically active experiments and abiotic controls. Therefore, unlike DCV and DRV, which showed consistent kinetic trends compared to total removal in abiotic controls, LOP and RIT showed strong affinity for adsorption onto the sludge surface in both cases. These observations are in accordance with our expectations, as both LOP and RIT are considered to be highly adsorbed onto porous surfaces such as activated sludge. The plateau observed for LOP removal is most likely related to the multicomponent solution and competitive adsorption, as previously mentioned, especially because all three of these specific kinetic trends cannot be fully separated. Therefore, we assume LOP was adsorbed in a constant amount onto the sludge surface, depending on the concentrations of other SASs, possibly in more than one layer, forming, to our knowledge, a “sandwich-like” sludge surface configuration.

### 3.4. Removal Efficiency Assessment

**Figure 5:**
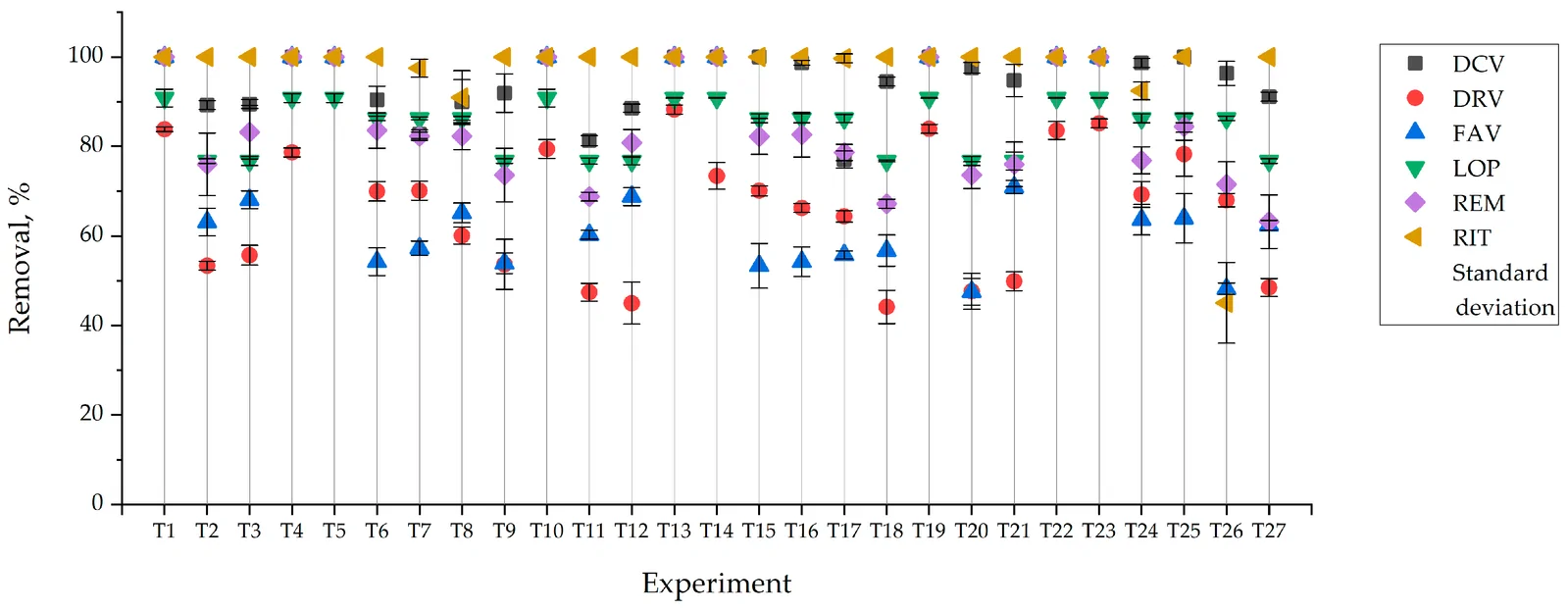
Removal efficiency of SASs after 48 h of treatment across all experimental runs of the FFD.

Bioaugmentation experiments showed distinct removal efficiencies after 48 h, depending on the bacterial inoculum, OD level, and SAS ratio in the mixture. Figure 5 shows overall SAS removal, categorized according to the experimental design labels of the FFD (T1–T27), whose conditions are listed in Table A1. All removal percentages ranged between 30–100%, with strong fluctuations depending on the experimental conditions. Although kinetic trends were similar for individual SAS removal, final concentrations varied significantly after 48 h.

RIT represented both the highest and lowest points on the plot and showed an especially high standard deviation in T26 (−1, −1, 1). As described in Section 3.3, this is assumed to be caused by desorption of RIT in the M1 ratio and inoculation with 0.1 OD of B3. DRV and FAV also showed high variability, with removal between 40–100%, indicating a strong influence of changing conditions on their removal. REM exhibited a removal range between 50–100%, while DCV and LOP showed removal between 70–100% across the FFD design.

The most efficient conditions according to the drop-line plot were T1 (−1, 1, −1), T5 (1, 1, −1), T10 (−1, 1, 0), T19 (−1, 1, 1), T22 (0, 1, 1), and T23 (1, 1, 1). These design experiments showed removal of above 80% for all SASs after 48 h of biological treatment. Common factors included a symmetrical SAS mix, M1, and the highest OD, while SAS-specific bacterial inoculation indicated an equal contribution to the process. These observations suggest that higher variability can be expected in experiments with higher concentrations of protease inhibitor SASs (M3), and also when one persistent SAS occurs at higher concentrations–in this case DRV, which was identified in our previous study [24] as a limiting factor for biodegradation due to its lowest removal (66%) even under optimal conditions, in contrast to other SASs (>90%).

### 3.5. Bioassay Evaluation

To evaluate the ecotoxicological effects of the bioaugmentation process, acute toxicity was assessed using the bioluminescent bacterium *Aliivibrio fischeri*. No detectable acute toxicity was observed in the treated aqueous phase after 48 h, with bioluminescence inhibition remaining close to 0% in both the bioaugmentation experiments and the corresponding controls. This finding differs markedly from our previous study [24], in which the investigated SASs, tested individually, caused substantial effects on A. fischeri, with inhibition values generally ranging from 30 to 60% before and after conventional activated-sludge biodegradation. The absence of measurable toxicity after bioaugmentation therefore indicates a substantial reduction in the bioavailable toxic fraction of the treated aqueous phase and suggests that potentially toxic transformation products, if formed, were not present at concentrations sufficient to induce an acute response in *A. fischeri*. Importantly, the absence of acute toxicity cannot be attributed solely to complete SAS removal, as several compounds remained detectable in the aqueous phase after 48 h. In addition to biodegradation and adsorption onto activated sludge, interactions with EPS produced by the inoculated bacteria (B1, B2, and B3) may have contributed to the reduced bioavailability of residual SASs. Binding or partitioning of these compounds within the EPS matrix could decrease the freely dissolved fraction available to *A. fischeri*, thereby reducing the observed toxic response without necessarily implying complete degradation of the parent compounds. This potential mechanism is further evaluated in relation to SAS removal kinetics, operational parameters, and the available literature.

### 3.6. RSM Modeling and Significant Factors Identification

For most of the investigated SASs (Table 7), the optimization responses were best described by a quadratic RSM model, indicating nonlinear relationships and curvature within the experimental design space. In contrast, the response for DCV followed a reduced two-factor interaction (2FI) model. This change in model type indicates that, although the individual factors had predominantly linear effects on this compound, the interaction between factors B and C was highly significant (*p* = 0.0095, Table A8). This distinct behavior can be attributed to the unique physicochemical properties of DCV, such as its specific hydrophobicity, which make its adsorption or biodegradation strictly dependent on the synergistic combination of the DCV ratio in a mixed solution and the type of bacterial inoculum. Although one data point (T17) was excluded from the DCV model, the biological trend was not disrupted. This outlier was identified using externally studentized residual diagnostics in Design-Expert 12. The exclusion of this single data point was based on model diagnostic analysis rather than on its absolute experimental value. Run T17 was identified as an outlier based on its externally studentized residual, indicating a substantial deviation from the response predicted by the fitted model. Its influence on model performance was further evaluated using the diagnostic tools implemented in Design-Expert. Following its exclusion, the residual distribution and overall model performance were improved, while the general experimental trend remained unchanged. Therefore, T17 was excluded to prevent a single statistically influential observation from disproportionately affecting model estimation and predictive performance. Additionally, the models for LOP and RIT were not applicable because of negative predicted R^2^ values. This was expected, since their removal percentages were tightly clustered, and the responses were better predicted using mean values of 64.61 ± 5.97% and 99.25 ± 2.29%, respectively. T26 for RIT model was also recognized as an outlier by the same steps as T17 for DCV.

All RSM models presented in Table 7 and Table A8 were significant (*p* < 0.05) with high *R*^2^ values (0.8142–0.9939), particularly for biological processes, and the differences between adjusted and predicted *R*^2^ wre acceptable (<0.2). Furthermore, adequate precision exceeded 4 for all models, particularly for REM (>20), indicating a good signal-to-noise ratio and supporting the use of the models to navigate the design space.

**Figure A2:**
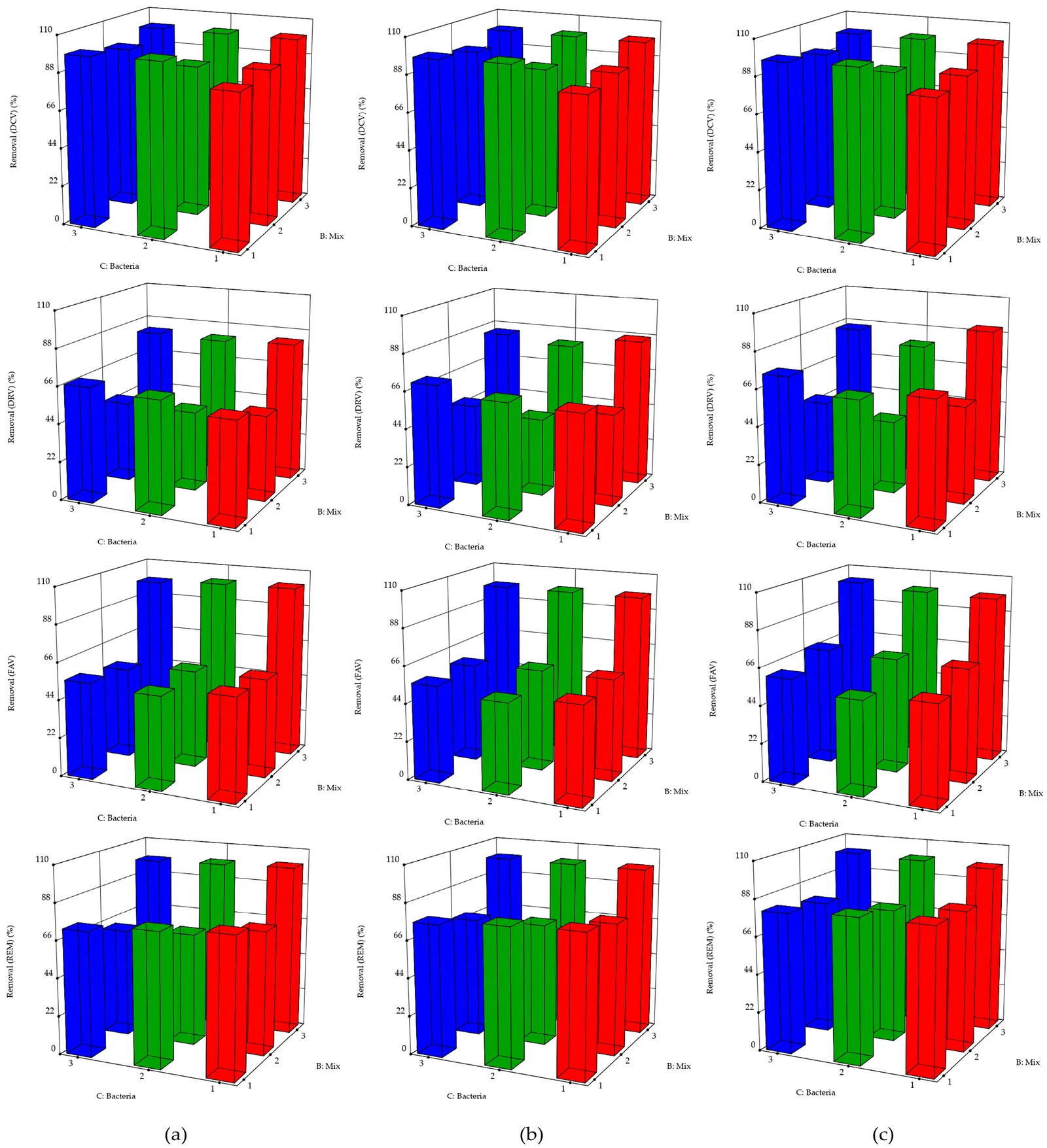
3D response plots showing the interactive effects of the experimental factors (red: bacteria B1; green: bacteria B2; blue: bacteria B3) on the removal efficiency of SASs for the three RSM models: (**a**) mixture M1; (**b**) mixture M2; and (**c**) mixture M3.

Instead of a single continuous 3D response surface, the relationships were visualized using 3D bar plots (Figure A2). This graphical representation was selected because the experimental design incorporated two categorical factors. Since categorical variables represent discrete groups rather than continuous numeric scales, the response cannot be modeled as a smooth surface, necessitating a distinct, compartmentalized 3D visualization for each category. Consequently, the final polynomial equations (Table A9) included the quadratic term exclusively for factor A (A^2^), as it was the only numeric (continuous) variable in the experimental design.

Overall, the most significant term was factor B (SAS mix ratio) for the DCV, DRV, FAV, and REM models. For DRV and FAV, it was the only significant term, whereas DCV and REM showed additional significant terms and interactions: C and BC for DCV, and A, C, and AB for REM. Factor A, as the only numerical factor, was relevant only for the REM model, including the A^2^ term. According to these observations, the removal efficiency was strongly and directly influenced by the multicomponent SAS concentrations and their interactions within the system, while the type of bacterial inoculum was significant only for the DCV and REM models. Furthermore, the significant interactions BC for DCV and AB for REM suggested that DCV removal was influenced by the SAS mix–bacteria relationship, whereas REM removal was additionally controlled by the SAS mix–OD relationship.

### 3.7. Process Optimization

To define the desirability of each factor in combination, specific criteria were established. SAS removal was set to its maximum efficiency, while categorical and numerical factors were constrained within the range of their investigated values (interval −1 to 1). By applying these criteria and using the RSM models, optimization solutions and predicted SAS removal were calculated and are presented in Table 8. Although 10 solutions were obtained, three had a desirability lower than 0.3 and were therefore not included with the other solutions.

The highest desirability (0.861) was achieved for M3 and B1, indicating a strong influence of B1 on SAS removal exceeding 84%, especially for protease inhibitor SAS, which were present at higher ratios in M3 (DRV, LOP, REM). The same bacterial inoculation type was identified as optimal for M2, with a desirability of 0.471 and a predicted removal exceeding 57%. In the symmetrical mix (equal ratio), B1 again proved to be the optimal bacterium, with a predicted removal exceeding 62% and a desirability of 0.574. Moreover, the optimal OD ranged from 0.993 to 1.000 for all selected solutions.

Accordingly, the optimal bacterial inoculation at the maximum tested OD was B1 in the solutions with higher concentrations of protease inhibitor SAS (M3) and equal SAS concentrations (M1), while B3 was identified as the optimal bacterium for bioaugmentation in the removal of the DRV-stress mix (DRV = 50%).

Although these solutions were chosen as the optimal conditions for each SAS mix, Table 8 also includes alternative choices with desirability higher than 0.4. These alternative conditions can be used to select similarly effective bacteria, such as B1, by accepting trade-offs in desirability and removal for slightly lower values, while significantly reducing bacterial maintenance costs within the system. Therefore, by choosing solutions with B1, the highest desirability was retained for M3 and M2, with trade-offs made for M1 by selecting the second alternative desirability of 0.469 and a removal of 59.30–99.25% instead of 62.17–99.25%.

It is also important to note that for M1 and M2, the predicted removal was lower than for M3, as DRV (49.36%) and FAV (59.30%) removal significantly limited the efficiency of the process within the system, while other SAS exhibited removal above 80% in all solutions. Interestingly, in M3, where DRV concentration was the same as LOP and RIT; and FAV concentration was the same as DCV and REM, an optimal balance among SAS was achieved, which enhanced their removal from the aqueous solution within 48 h. These observations in mixed solutions are valuable for controlling the bioaugmentation process in highly complex matrices typically present in wastewater treatment facilities.

## 4. Discussion

### 4.1. Properties of the Bacterial Inoculation System

Introducing specific bacteria into activated sludge is considered a promising approach for degrading recalcitrant and persistent micropollutants in wastewater systems [29]. Zhao et al. confirmed the successful implementation of bioaugmentation with *Rhodococcus* sp. strain p52 as a remediation strategy for treating multiple refractory pollutants, such as dibenzofuran, dibenzo-p-dioxin, and phenanthrene, which are known for their toxic environmental effects [30]. Significant results were also achieved by Ma et al. at 27–32 °C in the treatment of petrochemical wastewater, where the bioaugmented system reached the same effluent quality 10 days earlier than conventional activated sludge and showed stronger resistance to shock loadings, with a 5-day advantage [31]. Although research on the removal of SASs by bioaugmentation techniques remains limited, a few studies focus on the isolation and identification of SAS-degrading bacterial strains. The most notable studies include cultures of *Microcystis novacekii* and *Pseudomonas pseudoalcaligenes* for the removal of tenofovir disoproxil fumarate [32], and our published study on DCV, DRV, FAV, LOP, REM, RIT, and umifenovir removal by isolated SAS-tolerant cultures B1, B2, and B3 [27]. Michalska et al. also conducted an interesting study investigating suitable bioaugmentation candidates for leachate treatment and identifying *Pseudomonas putida* OR45a and KB3 as the most efficient bacteria, with a high capacity for degrading aromatic compounds [33].

Building on our previous studies, we investigated three bioaugmented systems, B1, B2, and B3, on selected SASs. A laboratory study by Boon et al. isolated B1 strain, examined its inoculation into activated sludge, and evaluated its ability to mineralize 3-chloroaniline [34]. Their results confirmed that this bacterial strain effectively degraded 3-chloroaniline within 2 weeks, while no degradation occurred in the non-inoculated control system. Furthermore, Kulaczkowski et al. demonstrated that the B1 bacterial strain was efficient in degrading microplastics, steroids, and complex organic pollutants as a reaction biocatalyst and suggested further studies on its inoculation into soil and wastewater [35]. The second strain investigated, B2, is considered a plant growth–promoting bacterium and is widely distributed in the environment. This species is known for producing various enzymes and exhibiting inhibitory effects on the growth of pathogens and polyketides, making it accessible and potentially highly efficient for bioremediation applications [36]. A laboratory study by Ndangang et al. isolated B2 from wastewater and identified significant in vitro enzymatic activities of this species, including catalase, amylase, and protease production, indicating promising potential for organic matter degradation in wastewater sludge [37]. The final inoculated culture, the B3 strain, was mainly investigated by Harinisri et al., who identified it as a potential bioflocculant and a sustainable alternative to conventional coagulants in wastewater treatment, improving activated sludge bulking and precipitation because of its specific filamentous morphology [38].

These bacterial strains, B1, B2, and B3, proved to be highly effective in individual SAS removal in our previous study [27], achieving over 90% removal, and showed high potential in present experiments on SAS mixtures and bioaugmentation of activated sludge. Although in all our previous research on SASs removal we used acclimated activated sludge with immediate exponential SAS removal or adsorption, in the present study the sludge was not pre-exposed, and distinct lag phases can be observed in Figure 4 during the initial 6 to 12 h, respectively. Rolfe et al. highlighted the lag phase as the most poorly understood stage of the bacterial growth cycle [39], while Hamill et al. determined that the length of the lag phase and growth rate inhibition, measured using model systems, are not reliable indicators of system stress [40]. Therefore, the delayed removal kinetics observed in Figure 4 cannot be directly linked to SAS-induced stress on the bacteria but are instead considered a phase of bacterial adjustment to the complex baseline system, activated sludge. Additionally, in the SASs mixture abiotic controls, potential competitive adsorption was observed, as expected for typical multicomponent pharmaceutical solutions [41], and will be discussed further in Section 4.3, SAS Classification According to Kinetic Trends.

### 4.2. Operational Parameters in Bioaugmentation Monitoring

The main objectives of bioaugmentation include increasing the density of desirable bacteria, increasing the overall organic removal rate, achieving specific operational goals, and recovering from upsets in the biological system. The effectiveness of the process in treatment plants is usually evaluated by monitoring:

- MLSS and MLVSS (daily), indicating changes in the quantity of microorganisms;
- Microscopic evaluation (every few days), indicating changes in microorganism consortia;
- Chemical oxygen demand, aiming for a steady increase in removal;
- Supernatant turbidity, measuring total suspended solids (TSS) and improvements in settling after 30 min;
- Sludge volume index, measuring the volume of settled sludge after 30 min (≤150 mL/g);
- Oxygen uptake rate, evaluating overflow leaving the bioreactor [42].

Using this technique to remove persistent organic micropollutants has several advantages, including preferential degradation of specific compounds, improved nitrification, better solids settling, enhanced COD removal, and overall healthier biomass [42,43]. In contrast, recurring limitations include death of the introduced microorganisms due to system stress or protozoan grazing, inoculum size effects, nutrient deficiencies, and bacteriophage infection [29].

#### 4.2.1. Influence of Basic Operational Parameters and Inoculum Concentrations

In the laboratory-scale bioaugmentation process, key operational parameters, including pH, temperature (*T*), dissolved oxygen (DO), electrical conductivity (EC), MLSS, MLVSS, water content, dry matter, and volatile matter, were monitored to characterize process conditions and support the interpretation of SAS removal performance [44]. In addition, CFU was determined for the raw activated sludge to establish the baseline bacterial count, and initial OD was determined to control bacterial inoculum density. Ramadan et al. investigated biodegradation of p-nitrophenol by *Pseudomonas cepacia* with CFU lower than 400 cells/mL and found that it was less effective than concentrations in the range of 10^4^–10^5^ cells/mL [45]. In these experiments, an initial OD of 0.1 was in the range of 10^6^–10^7^ cells/mL, 0.5 in the range of 10^7^–10^8^ cells/mL, and 1.0 reached 10^8^ cells/mL, irrespective of the bacterial inoculum type (B1, B2, B3). Activated sludge consortia reached the range of 10^6^ cells/mL, suggesting that the selected bacterial dosing concentrations were sufficient. During daily checks, all bacterial strains were effectively sustained over the 48 h test duration. Additionally, Ramadan et al. confirmed that low-density bacterial inoculum was not beneficial because of protozoan grazing, that is, protozoan growth through elimination of bacterial cultures. These findings directly correlate with our study, as the lower OD of 0.1 proved to be far less efficient than the highest OD of 1.0 for all SAS removals investigated.

#### 4.2.2. Monitoring of pH, EC, MLSS, and MLVSS Changes

Basic operational parameters were well controlled, as shown in Figure 3a. pH values remained constant throughout the experiment, whereas EC values increased after 24 h compared with the uninoculated controls. The increase in EC after 24 h can be linked to bacterial production of EPSs, which are considered a primary adaptive response of bacterial cultures to nutrient limitation and the presence of toxic substances [46]. Hu et al. examined the EC of pore water in soil after inoculation with a bacterial suspension and observed a rise in EC 10–19% as a result of bacterial secretion of ions and EPSs [47]. This correlates well with our previous research, as all SASs and their potential products were considered highly toxic to environmental microorganisms [5,24,48].

**Figure 6:**
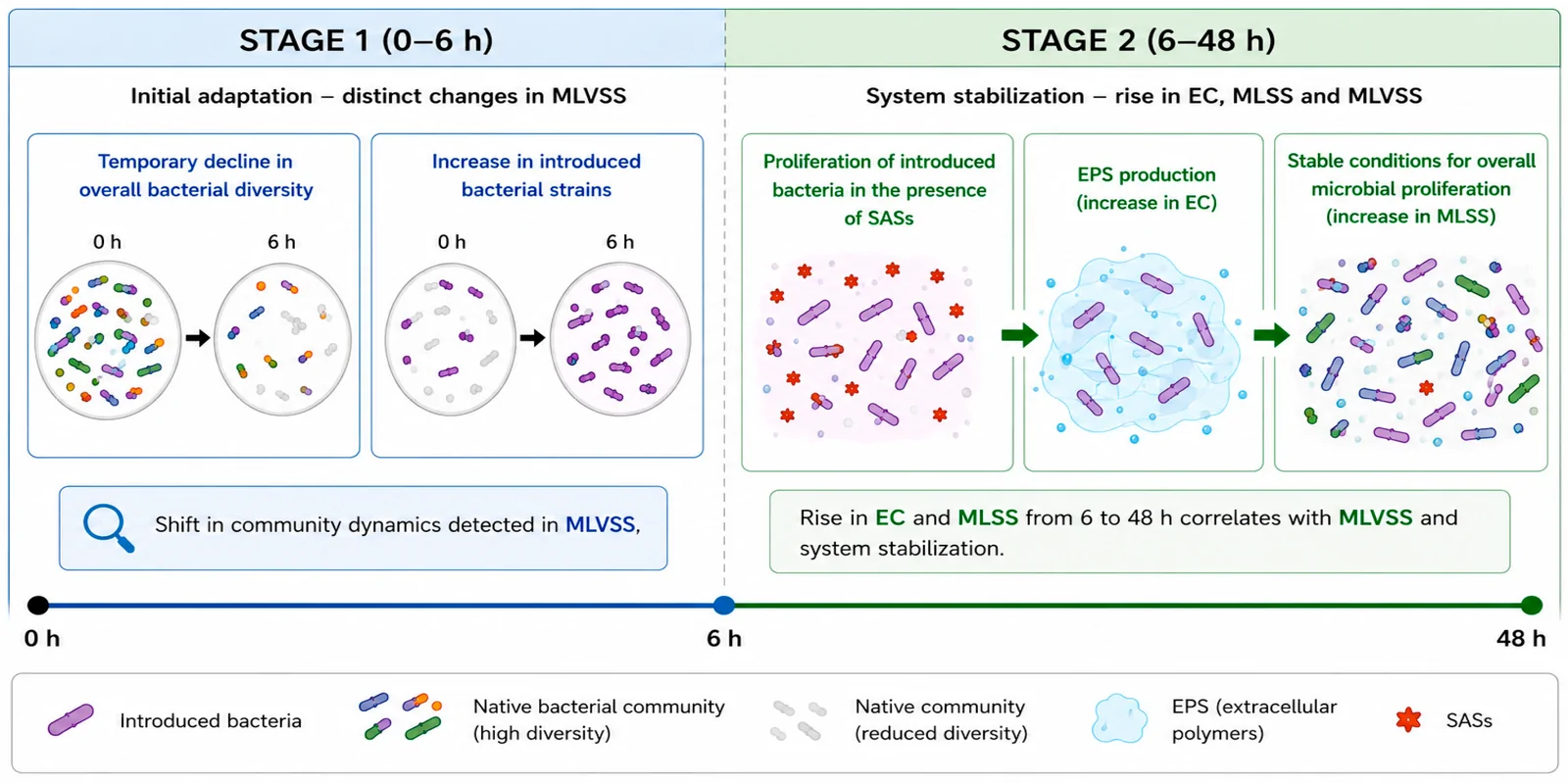
Schematic diagram of the process segmentation: Stage 1 (0–6 h), representing the initial adaptation phase; and Stage 2 (6–48 h), reflecting system stabilization, with highlighted dynamics of MLSS, MLVSS, and EC.

MLSS and MLVSS are shown in Figure 3b. The results were compared with uninoculated activated sludge controls, and trends for both experiments and their controls were similar and well correlated (Figure 6). The first stage included distinct changes in MLVSS at 6 h and may be related to a temporary decline in overall bacterial diversity and an increase in introduced bacterial strains [10], whose shift in community dynamics was not detected in MLSS. Consequently, in the second stage, the rise in EC and MLSS from 6 to 48 h directly correlates with MLVSS and system stabilization during this period, as elevated EC values can indicate EPS production caused by proliferation of introduced bacteria in the presence of toxins [46], as already discussed, and the resulting rise in MLSS values can indicate stable experimental conditions for overall microbial proliferation in the tested biological systems [44]. A significant contribution to the increase in EC and MLSS values could be associated with effective SAS mineralization and removal rates, which were also stabilized after 6 h for most investigated compounds (Figure 4).

#### 4.2.3. Discussion on Bacterial Activity at 45 °C

The experimental temperature of 45 °C may have contributed to the changes in microbial activity observed during the initial phase of the process. Previously identified as optimal for SAS biodegradation [24], this temperature lies near the upper range of mesophilic growth and may impose selective pressure on the activated sludge microbial community, favoring bacterial populations capable of maintaining metabolic activity under elevated-temperature conditions [44]. It directly affects the B1, B2, and B3 inoculum, whose temperature tolerance varies significantly. Although the MLSS, MLVSS, and EC showed similar trends, research on bacterial classification by temperature suggests otherwise. The BacDive database on bacterial diversity indicates that the B1, B2, and B3 strains do not have thermophilic properties, whereas research by Shafique et al. on enzyme activity in the *Bacillus* genus demonstrated different growth kinetics depending on temperature, with a significant increase at 45 °C [49]. Although the B1 strain was studied by Ghanbarinia et al., who determined that 45 °C may reduce bacterial activity and that maximal efficiency for lead uptake was detected at 37 °C [50], in this study there was no evidence of bacterial lysis or a decline in cell numbers. Furthermore, it was observed there was an average decline in DO levels compared to the controls, which indicates successful biodegradation by the introduced bacteria in the activated sludge.

Although the bacterial strains used for bioaugmentation are characteristically non-thermophilic, the operational temperature of 45 °C was deliberately selected based on the results of our previous optimization study [24], in which temperature was evaluated as one of the operational parameters affecting SAS removal by activated sludge. Within the investigated experimental design space, 45 °C provided the highest overall removal efficiency and was therefore selected for the present study to maintain consistency with the previously optimized conditions and to specifically evaluate the additional effect of bioaugmentation. Thus, the selection of 45 °C was based on process-level optimization of SAS removal rather than on the optimum growth temperature of the individual bacterial strains. It should also be recognized that 45 °C is close to the upper limit of the mesophilic range and may exert selective pressure on both the indigenous activated-sludge community and the introduced strains. Nevertheless, under the experimental conditions applied in the present study, no clear evidence of loss of functional stability was observed during the 48-h experimental period, as supported by the monitored MLSS, MLVSS, DO, and EC profiles. The use of 45 °C should therefore be interpreted as an experimentally optimized condition for the investigated laboratory-scale system rather than as a temperature representative of conventional wastewater treatment plants. Further studies under conventional wastewater-treatment temperatures are required to assess the transferability of the observed bioaugmentation effects to full-scale systems.

Considering that the experimental conditions applied in this study were selected for process optimization, it is important to distinguish the present approach from standardized OECD/ISO biodegradability testing. The present experiments were not designed to classify the investigated SASs in terms of ready or inherent biodegradability, as standardized tests use specifically defined conditions, endpoints, validity criteria, and test durations. Instead, the applied experimental design enabled the assessment of SAS removal from multicomponent mixtures and evaluation of the contribution of bioaugmentation under previously optimized conditions. The biodegradation behavior of the investigated SASs in activated sludge has already been addressed in our previous study [24], whereas the present work extends these findings by focusing on bioaugmentation and mixture-dependent removal.

Importantly, the observed decrease in parent-compound concentrations should be interpreted as removal rather than as direct evidence of ultimate biodegradation. Ultimate biodegradation implies mineralization of the organic compound and requires appropriate endpoints, such as oxygen consumption and/or CO_2_ production, as applied in standardized OECD/ISO biodegradation tests [51]. In the present study, DO was monitored as an indicator of the overall biological activity of the activated-sludge system; however, the observed oxygen depletion cannot be specifically attributed to the mineralization of individual SASs, and CO_2_ production was not quantified. Therefore, the reported removal efficiencies may reflect a combination of processes, including adsorption and biological transformation, rather than quantitatively demonstrated ultimate biodegradation. Standardized OECD/ISO biodegradation tests should therefore be regarded as a complementary approach for further verification of the biodegradation potential and mineralization of these compounds.

### 4.3. Classification According to Kinetic and Removal Trends

According to results presented in Figure 4 and Figure 5 for three mixtures, there were distinct trends observed and SAS classification into groups was presented as: (group 1) reversible adsorption (FAV, REM), (group 2) biodegradation (DCV, DRV), and (group 3) irreversible adsorption (LOP, RIT). A large number of studies is already published on these removal mechanisms on pharmaceuticals and are considered as common processes present in wastewater systems [52,53].

#### 4.3.1. Reversible Competitive Adsorption (Group 1)

FAV and REM exhibited reversible adsorption oscillation in experiments, which can generally be described as physical competition among different pharmaceuticals and dissolved or natural organic matter for the active sites of mesoporous surfaces, such as activated sludge or carbon [54]. Research by Mansouri et al. on competitive adsorption of ibuprofen and amoxicillin mixtures from aqueous solution onto activated carbon showed direct effects on equilibrium adsorption capacities, with higher uptake of ibuprofen and reduced adsorption of amoxicillin in complex water matrices compared with adsorption from individual pure solutions [55]. Their research also determined that specific interactions are controlled by pH, surface charge density, and pollutant ionization. Dubourg et al. investigated multicomponent effects on activated carbon and found that complex matrices decrease adsorption, especially in wastewater secondary effluents, where dissolved organic matter inhibits the adsorption rate of sulfamethoxazole and carbamazepine [56].

Xu et al. studied FAV and REM, as well as other SASs, under alkaline, high-cation conditions and found that FAV was mainly adsorbed via physisorption, whereas REM showed spontaneous sorption between physisorption and chemisorption [57]. Additional findings indicated that increased pH, cation strength and temperature decreased sorption capacities. This was also confirmed by our previous research on optimizing pH, temperature, and MLSS for the biodegradation process [24]. Observed kinetic trends in this research strongly support the study by Xu et al., although under different operational parameters, as FAV and REM showed high oscillation compared with the other investigated SASs, indicating that they bind to the outermost layer of the sludge surface, potentially through a combination of chemisorption and physisorption or solely through physisorption.

#### 4.3.2. Enhanced Bioavailability by EPS Production (Group 2)

Bioaugmentation of activated sludge in wastewater bioremediation involves significant matrix effects, particularly on the removal mechanisms of micropollutants. Deng et al. studied the biotransformation and adsorption of pharmaceuticals by activated sludge and evaluated the influence of matrix effects. High biotransformation efficiency was observed for diclofenac (52%), sulfadiazine, sulfamethoxazole, and roxithromycin (40%). In contrast, trimethoprim and carbamazepine showed no biotransformation potential [58]. In our previous study, we investigated and proposed potential removal mechanisms for selected SASs, also highlighting biotransformation potential in a matrix containing activated sludge for specific SASs [24]. Interestingly, DCV and DRV were not discussed as biotransformative SASs, but rather as compounds for which adsorption and biodegradation dominated, respectively.

The present findings further reveal the complex nature of SAS removal, as DCV and DRV showed variable results. It is important to consider EPS production when discussing these results, as its role in biological treatment processes could be highly significant in SAS removal. Huang et al. presented a review of the multiple roles of EPS in WWTPs. Their work highlighted that matrices rich in EPS critically influence microbial aggregates by changing adsorption capacity, stability, and formation [59]. A review by Melo et al. also emphasized the important role of EPS in micropollutant removal, identifying pH between 5.0 and 7.0 as ideal for EPS production, with maximum performance in the range of 25–31 °C [60]. Although our research was conducted at higher temperatures, the pH was ideal for EPS secretion, and retention time of 48 h was sufficient, which Melo et al. also identified as an important parameter. A review by Zhao et al. on EPS–antibiotic interactions in activated sludge determined that activated sludge flocs secrete EPS on their surface under antibiotic stress and, as a result, antibiotic diffusion was 40–72% lower than in water [61].

Although research on the influence of EPS on SASs is scarce and we cannot directly confirm high EPS production by inoculated bacteria based on our results, the trends we observed correlate with abundant CFU in the studied system and potential EPS production, which lowers DCV and DRV diffusion to the sludge flocs, decreases the adsorption rate, and increases bioavailability in the aqueous phase compared with denatured, inactivated sludge.

#### 4.3.3. Irreversible Adsorption and Initial Monolayer (Group 3)

Results obtained for LOP and RIT removal suggested strong, irreversible adsorption onto the surface of activated sludge and abiotic controls. Research by Krasucka et al. on the adsorption and desorption of LOP and RIT on sewage sludge highlighted significant competition between these drugs in binary solutions [18]. Both SASs were effectively adsorbed onto the sludge surface (80%), and their high adsorption distribution coefficients (*K*_d_ = 2076–3449 L/kg) were discussed as problematic once they are introduced into the environment as SAS–sludge combinations. In binary solution, lower adsorption of around 60–70% was observed for both drugs, which they attributed to a complex adsorption mechanism involving both physisorption and chemisorption.

**Figure 7:**
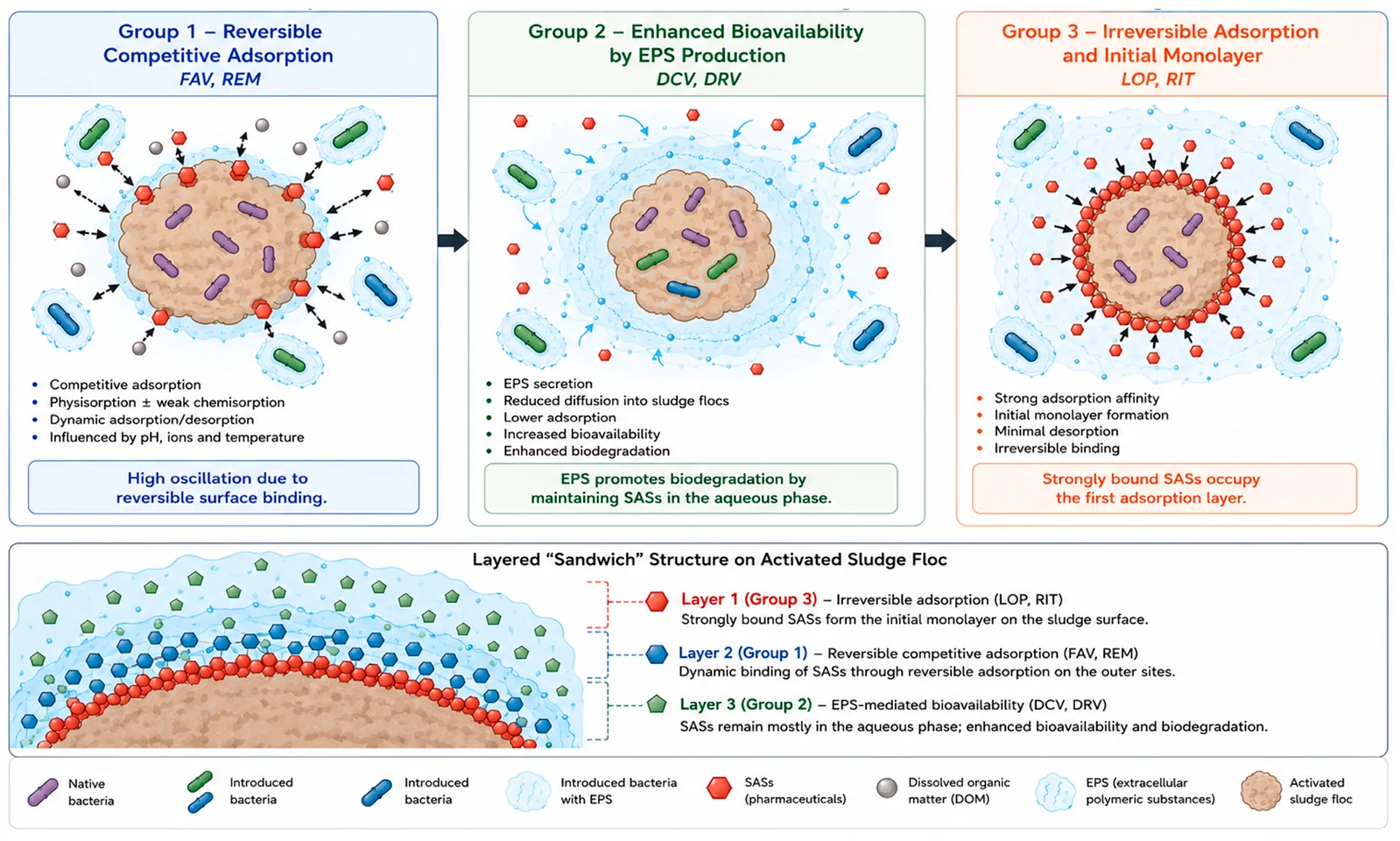
Proposed mechanisms of SASs removal in bioaugmented activated sludge, integrated with kinetic data and abiotic controls, highlighting the formation of a layered “sandwich” structure on the sludge surface.

This research is consistent with our findings, which indicate that LOP and RIT are hypothesized to preferentially occupy adsorption sites on activated sludge because of their extremely high adsorption affinities, followed by dynamic adsorption of the remaining medium- and low-bound SASs (Figure 7). This was particularly evident in the T1–T27 experiments, where RIT was completely adsorbed in both activated sludge and abiotic controls, while LOP remained at a constant removal level of 80–90%, which also correlates with the findings of Krasucka et al.

In our previous study, LOP was identified as an adsorption-dominated SAS, whereas RIT, although initially expected to exhibit similar behavior, showed substantial desorption from inactivated, uninoculated activated sludge after 24 h. In contrast, RIT exhibited a high biodegradation potential in biologically active sludge, indicating that its overall removal strongly depends on the biological activity of the system [24]. One exception was observed for the B3 (−1) inoculation test at 48 h (Figure 4a), indicating microbial destabilization and the release of a large amount of bound RIT from the sludge surface.

### 4.4. Matrix-Mediated Toxicity Suppression

In the toxicity assessment on *Aliivibrio fischeri*, no inhibitory effects were observed for the tested SASs mixtures. This finding can be linked to EPS production by the highly abundant bacterial CFU in the bioaugmented sludge. Melo et al. highlighted that EPS secretion is primarily used as a protective layer for bacterial cells against toxic compounds such as pharmaceuticals and pesticides. This protective ability of EPS is considered to be related to functional groups such as hydroxyl, carboxylic, sulfhydryl, and phosphate [60]. Lončarević et al. studied the toxicity of copper and two types of EPS (from *Bacillus licheniformis* and *Aureobasidium pullulans*) as potential protective agents in tests on *Daphnia magna* and *Aliivibrio fischeri*. Their research demonstrated a positive effect of EPS on the monitored respiration of *D. magna* and a reduced inhibition of *Aliivibrio fischeri* bioluminescence, with *EC*_10_ values shifting from 3.54 mg/L to 140.61 mg/L (EPS from *B. licheniformis*) and to 45.00 mg/L (EPS from *A. pullulans*) [62]. Although this research focused on copper ions, the presence of EPS shows a significant decrease in toxicity toward *Aliivibrio fischeri*, which, when combined with effective SASs removal by either adsorption or biodegradation, resulted in our bioaugmented sludge being non-toxic for all bacterial inocula due to masking effects of potentially high EPS production.

### 4.5. Implementation of RSM Models and Optimal Conditions

In our most recent study on individual SASs [24], the operating conditions for their biodegradation by uninoculated activated sludge were optimized and subsequently applied in the present study to evaluate and optimize the bioaugmentation process. In uninoculated activated sludge, removal efficiency reached 66% for DRV and exceeded 93% for the other individual SASs [24]. In the bioaugmented systems containing SAS mixtures, removal efficiencies generally exceeded 80%. Lower removal was observed for DRV (49.36%) and FAV (59.30%) under specific experimental conditions; however, under other combinations of bacterial strain, inoculum level, and SAS mixture composition, their removal efficiencies exceeded 90%, highlighting the strong influence of bioaugmentation conditions on the removal of these compounds.

RSM is considered an appropriate multifactorial modeling tool for optimizing various processes, such as adsorption or removal of pharmaceuticals in different systems [63,64]. FFD is also commonly used in combination with RSM to optimize degradation kinetics, as investigated by Juretić et al. for photooxidation of benzene-structured compounds with three parameters at three levels [65]. A major advantage of FFD compared with other designs is its superior resolution for individual factors; however, it is generally modeled with linear regressions and requires a large number of experiments [66]. Furthermore, Aydar et al. highlighted that RSM enables evaluation of the effects of multiple factors and their interactions on multiple response variables and plays an important role in several scientific areas [67]. A review by Karimifard et al. stated that parameters determined as statistically significant by ANOVA are unlikely to occur by chance. The occurrence probability of a given event is represented by the *p*-value, which is lower than 0.05 for significant factors. They also highlighted RSM limitations related to inaccurate extrapolation of results outside experimental ranges; however, modeling with this methodology still has many positive features [68].

The desirability function ranges from 0 (outside the limits) to 1 (goal) and represents a mathematical method used to find the optimum. Stat-Ease, the provider of Design-Expert software, also emphasized that the optimization goal is not to achieve the highest desirability level, but to determine a set of conditions that meet all defined goals [69]. Therefore, it was considered all acceptable solutions calculated by the software (Table 8), analyzed predicted SAS removal, and determined B1 as the optimal inoculation type, an OD of 1.0 as the optimal dosing, and M1 (D = 0.469), M2 (D = 0.471), and M3 (D = 0.861) as the optimal mixture settings.

The most significant factor was the SAS ratios in mixtures, which were expected to be dominant. As already discussed in Section 4.3, Classification According to Kinetic and Removal Trends, different ratios exhibited competitive adsorption onto the sludge surface, and distinct bioavailability was achieved for each mixture, leading to variable removal of SASs depending on their mutual interaction in the aqueous phase and at active sites on the activated sludge.

In conventional WWTPs polinomical equations received through RSM modeling can be used in pilot-plants which exhibited in a study by Nghia et al. a deviation of around 2% in real wastewater compared to predicted dye removal value [70]. In contrast, research from 2025 by Shaaban et al. highlighted artificial intelligence (AI) is becoming extensively used in operation and management of WWTPs and can be applied in combination to RSM predicted models as a powerful tool for predicting and optimizing efficiency in wastewater removal processes [71].

## 5. Conclusions

This study provides new insights into the removal behavior of SASs in bioaugmented activated sludge under laboratory-controlled conditions. The combined application of FFD and RSM revealed nonlinear removal behavior and identified SAS mixture composition as a major factor governing process efficiency. Based on their characteristic kinetic and adsorption profiles, the investigated SASs were classified into three groups: reversible sorption (FAV and REM), enhanced aqueous-phase bioavailability (DCV and DRV) and strong, poorly reversible sorption (LOP and RIT). These distinct behaviors demonstrate that interactions among pharmaceuticals in multicomponent systems can substantially affect their fate and removal in activated sludge.

Bioaugmentation supported high removal efficiencies for most SASs, although DRV and FAV remained the most challenging compounds under specific experimental conditions. The high abundance of culturable bacteria and the absence of detectable acute toxicity toward *Aliivibrio fischeri* after treatment indicate favorable biological activity and environmental performance of the bioaugmented system. Microbially mediated changes in the sludge matrix, potentially involving EPS, may have altered SAS partitioning and reduced the bioavailable toxic fraction, although this mechanism requires further investigation. Overall, integrating kinetic removal data, adsorption behavior, ecotoxicity assessment, and RSM modeling provides a comprehensive framework for understanding and optimizing SAS removal. Future integration of validated RSM models with artificial intelligence-based predictive approaches could further support process optimization and scale-up toward conventional wastewater treatment applications.

## Figures and Tables

**Table 1:** Composition of the mineral medium per liter [25].

Components	Mass, g
K_2_HPO_4_	12.8
KH_2_PO_4_	1.1
(NH_4_)_2_SO_4_	1.0
MgSO_4_·7 H_2_O	0.1
+ Trace elements solution	5.0 mL

Included trace elements were: H_3_BO_3_, ZnSO_4_·6 H_2_O, FeSO_4_(NH_4_)_2_SO_4_·6 H_2_O, CoSO_4_·7 H_2_O, (NH_4_)_6_Mo_7_O_24_·4 H_2_O, CuSO_4_·5 H_2_O, and MnSO_4_·4 H_2_O.

**Table 2:** List of SASs concentrations used for preparation of three distinct mix solutions (M1, M2, M3) for bioaugmentation experiments.

Mix (FFD)	SASs Ratio	DCV, μmol/L	DRV, μmol/L	FAV, μmol/L	LOP, μmol/L	REM, μmol/L	RIT, μmol/L
M1 (1)	Symmetrical mixture	1.00	1.00	1.00	1.00	1.00	1.00
M2 (2)	DRV-enriched mixture	0.60	3.00	0.60	0.60	0.60	0.60
M3 (3)	Protease inhibitor-enriched mixture	0.50	1.50	0.50	1.50	0.50	1.50

FFD: Full factorial design.

**Table 3:** Coded values of numerical and categorical factors in the full factorial design (FFD) for the bioaugmentation process.

Factor A: OD		Factor B: Mix		Factor C: Bacteria	
Level	Value	Level	Value	Level	Value
−1	0.1	−1	1	−1	1
0	0.5	0	2	0	2
1	1.0	1	3	1	3

**Table 4:** Optimized HPLC parameters including flow rate, mobile phase ratio, injection volume, and column temperature.

Time, min	%B
0.00	15.0
2.50	15.0
5.00	55.0
10.00	55.0
10.01	15.0
15.00	10.0
Flow rate, mL/min	0.250
Injection volume, μm	10.0
*T*, °C	40.0

**Table 5:** Analytical performance and calibration parameters of the developed HPLC method.

Condition	DCV	DRV	FAV	LOP	REM	RIT
Wavelength, nm	315	260	315	205	241	241
*t*_R_, min	6.350	8.568	2.547	11.347	10.567	8.050
*R* ^2^	0.9948	0.9957	0.9972	0.9984	0.9940	0.9986
LOD, μmol/L	0.030	0.027	0.026	0.023	0.032	0.016
LOQ, μmol/L	0.089	0.081	0.077	0.069	0.096	0.047

*t*_R_: Retention time; *R*^2^: Coefficient of determination; LOD: Limit of detection; LOQ: Limit of quantification.

**Table 6:** Physicochemical and microbiological properties of activated sludge used in the experiments.

Parameter	Value
pH	7.35 ± 0.73
*T*, °C	23.0 ± 0.4
DO, mg/L	5.23 ± 1.82
EC, mS/cm	0.68 ± 0.15
*w* (H_2_O), %	99.31 ± 0.14
*w* (dry matter), %	0.69 ± 0.14
*w* (volatile matter), %	69.43 ± 2.71
MLSS, g/L	3.50 ± 0.58
MLVSS, %	82.19 ± 7.00
CFU (activated sludge), cells/mL	(4.05 ± 0.52) × 10^6^

Values represent mean ± SD (*n* = 3).

**Table 7:** Fit statistics and adequacy summary of the RSM models.

Response	Model Type	*R* ^2^	Adjusted *R*^2^	Predicted *R*^2^	Adeq Pecision	*p*-Value
DCV removal, %	Reduced 2FI	0.8142	0.7097	0.5327	6.9629	0.0002
DRV removal, %	Quadratic	0.9484	0.8882	0.7334	12.7904	<0.0001
FAV removal, %	Quadratic	0.9618	0.9173	0.8097	11.3460	<0.0001
REM removal, %	Quadratic	0.9839	0.9651	0.9066	22.1434	<0.0001

Design-Expert 12 package was used for modeling of data.

**Table 8:** Model prediction, desirability function, and process potential metrics for SASs removal.

A: OD	B: Mix	C: Bacteria	Predicted SAS Removal	Desirability	Process Potential
1.000	3	1	84.61–99.45%	0.861	Optimal M3 conditions
0.993	3	2	79.23–100.00%	0.824	Alternative M3 conditions
1.000	1	3	62.17–99.25%	0.574	Optimal M1 conditions
1.000	1	2	55.57–99.25%	0.510	Alternative M1 conditions
1.000	2	1	57.63–99.25%	0.471	Optimal M2 conditions
1.000	1	1	59.30–99.25%	0.469	Second M1 alternative
1.000	2	3	49.37–99.25%	0.420	Alternative M2 conditions

Design-Expert 12 package was used for modeling of data.

## Data Availability

The original contributions presented in this study are included in the article. Further inquiries can be directed to the corresponding author.

## Appendix A

**Table A1:** Full factorial design (FFD, 3^3^) for the bioaugmentation process.

Experiment	Run	Factor A: OD		Factor B: Mix		Factor C: Bacteria	
		Level	Value	Level	Value	Level	Value
T1	7	−1	0.1	1	3	−1	1
T2	5	0	0.5	0	2	−1	1
T3	6	1	1.0	0	2	−1	1
T4	8	0	0.5	1	3	−1	1
T5	9	1	1.0	1	3	−1	1
T6	2	0	0.5	−1	1	−1	1
T7	3	1	1.0	−1	1	−1	1
T8	1	−1	0.1	−1	1	−1	1
T9	4	−1	0.1	0	2	−1	1
T10	16	−1	0.1	1	3	0	2
T11	14	0	0.5	0	2	0	2
T12	15	1	1.0	0	2	0	2
T13	17	0	0.5	1	3	0	2
T14	18	1	1.0	1	3	0	2
T15	11	0	0.5	−1	1	0	2
T16	12	1	1.0	−1	1	0	2
T17	10	−1	0.1	−1	1	0	2
T18	13	−1	0.1	0	2	0	2
T19	25	−1	0.1	1	3	1	3
T20	23	0	0.5	0	2	1	3
T21	24	1	1.0	0	2	1	3
T22	26	0	0.5	1	3	1	3
T23	27	1	1.0	1	3	1	3
T24	20	0	0.5	−1	1	1	3
T25	21	1	1.0	−1	1	1	3
T26	19	−1	0.1	−1	1	1	3
T27	22	−1	0.1	0	2	1	3

**Table A2:** Initial OD measurements at *λ* = 600 nm prior to mixing with activated sludge for experimental setup.

Exp	OD (*λ* = 600 nm)		
	M1	M2	M3
T1	0.13 ± 0.03	0.14 ± 0.03	0.09 ± 0.03
T2	0.43 ± 0.03	0.47 ± 0.09	0.49 ± 0.05
T3	1.04 ± 0.08	1.02 ± 0.09	0.98 ± 0.09
T4	0.44 ± 0.04	0.47 ± 0.04	0.51 ± 0.04
T5	1.02 ± 0.03	1.04 ± 0.03	0.99 ± 0.03
T6	0.44 ± 0.04	0.49 ± 0.04	0.52 ± 0.04
T7	1.04 ± 0.07	1.03 ± 0.07	1.01 ± 0.09
T8	0.15 ± 0.02	0.15 ± 0.00	0.11 ± 0.02
T9	0.12 ± 0.01	0.12 ± 0.01	0.13 ± 0.01
C1 (B1, −1)	0.13 ± 0.02	0.12 ± 0.02	0.10 ± 0.02
C2 (B1, 0)	0.46 ± 0.01	0.48 ± 0.01	0.46 ± 0.07
C3 (B1, 1)	1.01 ± 0.10	1.00 ± 0.01	0.99 ± 0.08
T10	0.13 ± 0.01	0.11 ± 0.00	0.12 ± 0.01
T11	0.63 ± 0.08	0.49 ± 0.08	0.63 ± 0.08
T12	1.08 ± 0.04	1.01 ± 0.09	1.03 ± 0.04
T13	0.59 ± 0.07	0.52 ± 0.07	0.65 ± 0.07
T14	1.05 ± 0.01	1.04 ± 0.05	1.03 ± 0.10
T15	0.57 ± 0.06	0.51 ± 0.06	0.63 ± 0.06
T16	1.08 ± 0.03	1.02 ± 0.03	1.05 ± 0.03
T17	0.14 ± 0.00	0.14 ± 0.01	0.15 ± 0.01
T18	0.17 ± 0.02	0.13 ± 0.02	0.13 ± 0.02
C1 (B2, −1)	0.14 ± 0.02	0.10 ± 0.02	0.12 ± 0.02
C2 (B2, 0)	0.55 ± 0.03	0.60 ± 0.03	0.58 ± 0.03
C3 (B2, 1)	1.10 ± 0.10	1.05 ± 0.05	1.08 ± 0.03
T19	0.14 ± 0.05	0.15 ± 0.08	0.15 ± 0.01
T20	0.46 ± 0.06	0.56 ± 0.06	0.46 ± 0.06
T21	0.92 ± 0.05	1.01 ± 0.09	0.95 ± 0.05
T22	0.48 ± 0.06	0.57 ± 0.06	0.46 ± 0.06
T23	0.94 ± 0.03	0.98 ± 0.03	0.92 ± 0.03
T24	0.45 ± 0.07	0.56 ± 0.07	0.44 ± 0.02
T25	0.93 ± 0.04	0.99 ± 0.04	0.98 ± 0.04
T26	0.14 ± 0.01	0.16 ± 0.012	0.15 ± 0.00
T27	0.15 ± 0.01	0.14 ± 0.01	0.14 ± 0.07
C1 (B3, −1)	0.15 ± 0.06	0.15 ± 0.01	0.14 ± 0.00
C2 (B3, 0)	0.49 ± 0.05	0.55 ± 0.05	0.45 ± 0.05
C3 (B3, 1)	0.13 ± 0.03	0.14 ± 0.03	0.09 ± 0.09

Exp: Experiment; M: Mix, B: Bacteria; OD: Optical density.

**Table A3:** Measured pH at 0 h, 6 h, 24 h and 48 h for B1, B2, and B3.

Exp	pH, −			
	0 h	6 h	24 h	48 h
T1	6.08 ± 0.07	6.21 ± 0.04	6.25 ± 0.05	6.43 ± 0.02
T2	6.08 ± 0.06	6.21 ± 0.01	6.25 ± 0.06	6.43 ± 0.02
T3	5.90 ± 0.09	6.03 ± 0.03	6.14 ± 0.06	6.19 ± 0.00
T4	5.91 ± 0.09	6.03 ± 0.04	6.15 ± 0.06	6.19 ± 0.04
T5	5.95 ± 0.01	6.03 ± 0.07	6.17 ± 0.09	6.29 ± 0.06
T6	6.03 ± 0.08	6.18 ± 0.03	6.38 ± 0.07	6.31 ± 0.03
T7	6.03 ± 0.09	6.22 ± 0.01	6.41 ± 0.07	6.34 ± 0.03
T8	6.05 ± 0.02	6.09 ± 0.06	6.41 ± 0.04	6.22 ± 0.08
T9	5.92 ± 0.03	6.07 ± 0.06	6.16 ± 0.05	6.35 ± 0.02
T10	5.96 ± 0.04	6.09 ± 0.04	6.19 ± 0.08	6.34 ± 0.05
T11	5.96 ± 0.03	6.09 ± 0.01	6.19 ± 0.09	6.34 ± 0.02
T12	5.99 ± 0.02	6.15 ± 0.02	6.11 ± 0.07	6.15 ± 0.01
T13	5.99 ± 0.05	6.15 ± 0.00	6.10 ± 0.07	6.14 ± 0.01
T14	6.06 ± 0.09	6.16 ± 0.09	6.25 ± 0.03	6.42 ± 0.06
T15	6.04 ± 0.02	6.23 ± 0.06	6.37 ± 0.09	6.31 ± 0.08
T16	6.05 ± 0.03	6.35 ± 0.05	6.38 ± 0.04	6.31 ± 0.07
T17	6.02 ± 0.09	6.16 ± 0.04	6.32 ± 0.06	6.29 ± 0.04
T18	5.95 ± 0.01	6.03 ± 0.07	6.17 ± 0.03	6.29 ± 0.06
T19	6.02 ± 0.03	6.12 ± 0.04	6.22 ± 0.07	6.38 ± 0.02
T20	6.02 ± 0.03	6.12 ± 0.06	6.22 ± 0.07	6.38 ± 0.03
T21	5.99 ± 0.08	6.01 ± 0.05	6.25 ± 0.03	6.42 ± 0.06
T22	5.99 ± 0.06	6.01 ± 0.04	6.24 ± 0.02	6.42 ± 0.05
T23	5.99 ± 0.05	6.01 ± 0.04	6.24 ± 0.03	6.45 ± 0.06
T24	6.05 ± 0.05	6.36 ± 0.02	6.28 ± 0.06	6.35 ± 0.03
T25	6.07 ± 0.08	6.41 ± 0.05	6.47 ± 0.02	6.40 ± 0.07
T26	6.01 ± 0.02	6.34 ± 0.06	6.31 ± 0.04	6.29 ± 0.09
T27	6.06 ± 0.04	6.16 ± 0.08	6.25 ± 0.01	6.42 ± 0.06
C1 (M1)	6.05 ± 0.04	6.06 ± 0.08	6.38 ± 0.01	6.21 ± 0.06
C2 (M2)	6.03 ± 0.05	6.13 ± 0.08	6.10 ± 0.03	6.38 ± 0.08
C3 (M3)	6.03 ± 0.05	6.13 ± 0.08	6.10 ± 0.01	6.38 ± 0.08

Values are presented as mean ± SD (*n* = 3). Exp: Experimental run; C: Uninoculated sludge control 1 (MIX 1); control 2 (MIX 2) control 3 (MIX 3).

**Table A4:** Measured DO at 0 h, 6 h, 24 h and 48 h for B1, B2, and B3.

Exp	DO, mg/L			
	0 h	6 h	24 h	48 h
T1	2.19 ± 0.44	2.03 ± 0.23	4.23 ± 0.25	2.73 ± 0.17
T2	2.18 ± 0.43	3.36 ± 0.18	4.40 ± 0.38	4.18 ± 0.37
T3	3.00 ± 0.50	3.93 ± 0.29	4.45 ± 0.39	3.28 ± 0.42
T4	2.21 ± 0.43	3.41 ± 0.17	3.32 ± 0.49	3.26 ± 0.38
T5	2.06 ± 0.34	2.70 ± 0.30	2.88 ± 0.38	3.72 ± 0.20
T6	5.47 ± 0.52	5.69 ± 0.55	4.62 ± 0.39	3.83 ± 0.32
T7	5.66 ± 0.48	4.42 ± 0.36	5.40 ± 0.41	3.08 ± 0.58
T8	5.55 ± 0.59	6.05 ± 0.59	5.50 ± 0.34	4.33 ± 0.35
T9	4.24 ± 0.26	3.64 ± 0.25	4.30 ± 0.30	4.22 ± 0.49
T10	2.07 ± 0.36	2.04 ± 0.35	4.30 ± 0.43	4.30 ± 0.24
T11	2.85 ± 0.47	1.89 ± 0.22	3.80 ± 0.48	5.80 ± 0.39
T12	2.50 ± 0.11	2.25 ± 0.39	2.98 ± 0.33	5.66 ± 0.48
T13	2.54 ± 0.48	1.84 ± 0.13	3.14 ± 0.45	3.64 ± 0.32
T14	1.93 ± 0.46	1.76 ± 0.40	2.44 ± 0.18	3.03 ± 0.30
T15	4.84 ± 0.35	2.50 ± 0.36	5.00 ± 0.54	4.03 ± 0.54
T16	4.55 ± 0.32	1.60 ± 0.39	3.86 ± 0.36	3.88 ± 0.39
T17	5.80 ± 0.35	4.25 ± 0.35	3.90 ± 0.44	3.96 ± 0.59
T18	3.63 ± 0.20	2.38 ± 0.16	5.46 ± 0.38	6.04 ± 0.46
T19	2.35 ± 0.34	4.78 ± 0.45	3.44 ± 0.42	2.96 ± 0.48
T20	2.82 ± 0.28	5.40 ± 0.43	4.10 ± 0.50	5.51 ± 0.33
T21	2.25 ± 0.34	5.19 ± 0.27	3.31 ± 0.46	5.50 ± 0.28
T22	2.00 ± 0.17	3.30 ± 0.14	2.61 ± 0.35	3.37 ± 0.25
T23	2.95 ± 0.36	2.80 ± 0.37	2.12 ± 0.29	2.35 ± 0.37
T24	4.96 ± 0.48	4.99 ± 0.43	4.47 ± 0.48	3.83 ± 0.51
T25	4.40 ± 0.51	3.38 ± 0.39	4.37 ± 0.31	3.53 ± 0.43
T26	5.16 ± 0.56	5.25 ± 0.46	4.99 ± 0.45	3.46 ± 0.33
T27	3.40 ± 0.40	4.68 ± 0.18	4.79 ± 0.48	5.15 ± 0.44
C1 (M1)	5.70 ± 0.41	5.63 ± 0.31	4.54 ± 0.48	5.35 ± 0.48
C2 (M2)	3.60 ± 0.48	4.70 ± 0.49	4.75 ± 0.43	3.60 ± 0.13
C3 (M3)	2.48 ± 0.33	3.45 ± 0.36	5.85 ± 0.22	3.47 ± 0.11

Values are presented as mean ± SD (*n* = 3). Exp: Experimental run; C: Uninoculated sludge control 1 (MIX 1); control 2 (MIX 2) control 3 (MIX 3).

**Table A5:** Measured EC at 0 h, 6 h, 24 h and 48 h for B1, B2, and B3.

Exp	EC, mS/cm			
	0 h	6 h	24 h	48 h
T1	10.96 ± 0.35	11.34 ± 0.34	12.23 ± 0.45	14.81 ± 0.40
T2	10.52 ± 0.29	10.71 ± 0.26	12.57 ± 0.24	15.96 ± 0.39
T3	10.02 ± 0.28	10.18 ± 0.12	11.67 ± 0.26	13.31 ± 0.40
T4	10.72 ± 0.17	10.79 ± 0.16	11.40 ± 0.27	12.52 ± 0.25
T5	10.31 ± 0.33	10.58 ± 0.16	11.38 ± 0.37	12.59 ± 0.29
T6	10.16 ± 0.33	10.23 ± 0.20	8.86 ± 0.29	10.32 ± 0.13
T7	10.11 ± 0.37	10.20 ± 0.21	10.64 ± 0.31	12.10 ± 0.19
T8	10.19 ± 0.18	10.30 ± 0.30	11.41 ± 0.23	13.19 ± 0.12
T9	9.86 ± 0.39	10.28 ± 0.27	10.90 ± 0.49	11.79 ± 0.37
T10	10.42 ± 0.33	11.02 ± 0.36	11.64 ± 0.39	11.97 ± 0.37
T11	10.16 ± 0.35	10.26 ± 0.44	11.41 ± 0.39	13.07 ± 0.34
T12	10.18 ± 0.39	10.60 ± 0.23	11.30 ± 0.24	12.51 ± 0.19
T13	10.53 ± 0.17	11.08 ± 0.15	11.81 ± 0.20	12.51 ± 0.10
T14	10.62 ± 0.24	11.20 ± 0.16	11.85 ± 0.32	13.68 ± 0.44
T15	10.19 ± 0.24	10.34 ± 0.19	12.69 ± 0.40	14.88 ± 0.13
T16	10.16 ± 0.27	10.38 ± 0.24	12.35 ± 0.36	16.43 ± 0.11
T17	10.18 ± 0.42	10.28 ± 0.25	11.06 ± 0.16	11.99 ± 0.30
T18	10.14 ± 0.17	10.29 ± 0.17	11.06 ± 0.31	12.72 ± 0.31
T19	10.68 ± 0.18	10.81 ± 0.15	13.82 ± 0.19	15.69 ± 0.49
T20	10.17 ± 0.27	10.27 ± 0.30	13.66 ± 0.27	12.69 ± 0.22
T21	10.03 ± 0.27	10.27 ± 0.46	11.66 ± 0.48	11.72 ± 0.30
T22	10.60 ± 0.44	10.86 ± 0.31	12.29 ± 0.27	16.59 ± 0.17
T23	10.42 ± 0.22	10.89 ± 0.35	12.24 ± 0.24	15.03 ± 0.39
T24	10.15 ± 0.22	10.46 ± 0.23	11.73 ± 0.39	14.30 ± 0.13
T25	10.08 ± 0.34	10.32 ± 0.27	13.10 ± 0.19	15.94 ± 0.35
T26	10.18 ± 0.34	10.30 ± 0.21	11.94 ± 0.31	14.74 ± 0.16
T27	10.30 ± 0.22	10.56 ± 0.35	12.00 ± 0.14	12.32 ± 0.27
C1 (M1)	10.09 ± 0.22	10.33 ± 0.41	11.47 ± 0.14	13.13 ± 0.37
C2 (M2)	10.21 ± 0.38	10.32 ± 0.21	11.04 ± 0.19	12.70 ± 0.32
C3 (M3)	10.23 ± 0.20	10.51 ± 0.17	10.73 ± 0.41	11.21 ± 0.42

Values are presented as mean ± SD (*n* = 3). Exp: Experimental run; C: Uninoculated sludge control 1 (MIX 1); control 2 (MIX 2) control 3 (MIX 3).

**Table A6:** Measured MLSS at 0 h, 6 h, 24 h and 48 h for B1, B2, and B3.

Exp	MLSS, g/L			
	0 h	6 h	24 h	48 h
T1	3.56 ± 0.18	5.04 ± 0.39	5.44 ± 0.44	6.58 ± 0.47
T2	4.02 ± 0.25	2.36 ± 0.12	4.78 ± 0.32	5.02 ± 0.38
T3	3.76 ± 0.26	3.48 ± 0.24	3.74 ± 0.27	4.30 ± 0.30
T4	4.26 ± 0.32	4.78 ± 0.37	5.78 ± 0.46	5.88 ± 0.40
T5	3.84 ± 0.37	3.90 ± 0.38	4.38 ± 0.37	4.64 ± 0.46
T6	3.68 ± 0.24	3.86 ± 0.24	3.64 ± 0.25	4.54 ± 0.34
T7	3.98 ± 0.27	2.76 ± 0.25	3.26 ± 0.31	3.24 ± 0.30
T8	3.76 ± 0.25	3.56 ± 0.22	4.10 ± 0.26	4.54 ± 0.30
T9	4.06 ± 0.28	3.76 ± 0.23	4.08 ± 0.30	4.68 ± 0.27
T10	4.10 ± 0.29	4.90 ± 0.36	4.86 ± 0.32	5.00 ± 0.40
T11	4.10 ± 0.27	3.96 ± 0.25	3.96 ± 0.29	4.82 ± 0.32
T12	4.30 ± 0.29	3.00 ± 0.16	5.48 ± 0.38	6.04 ± 0.41
T13	3.52 ± 0.25	4.04 ± 0.30	5.20 ± 0.35	5.20 ± 0.40
T14	4.18 ± 0.40	3.86 ± 0.32	4.68 ± 0.40	5.20 ± 0.45
T15	3.62 ± 0.24	3.66 ± 0.24	4.24 ± 0.30	5.34 ± 0.41
T16	4.12 ± 0.28	4.12 ± 0.31	5.24 ± 0.41	8.06 ± 0.57
T17	3.60 ± 0.23	2.44 ± 0.14	4.50 ± 0.32	4.26 ± 0.32
T18	4.42 ± 0.28	3.86 ± 0.25	4.32 ± 0.28	3.66 ± 0.22
T19	3.82 ± 0.32	3.28 ± 0.28	4.74 ± 0.43	6.40 ± 0.52
T20	3.60 ± 0.33	2.92 ± 0.25	4.60 ± 0.44	4.56 ± 0.42
T21	4.34 ± 0.31	3.98 ± 0.26	4.74 ± 0.32	3.94 ± 0.28
T22	4.12 ± 0.50	3.22 ± 0.34	3.64 ± 0.41	6.68 ± 0.72
T23	4.42 ± 0.40	5.92 ± 0.51	7.24 ± 0.62	8.46 ± 0.75
T24	3.96 ± 0.27	3.30 ± 0.21	4.70 ± 0.31	5.26 ± 0.35
T25	4.30 ± 0.30	3.40 ± 0.21	5.46 ± 0.37	9.64 ± 0.78
T26	3.88 ± 0.26	3.84 ± 0.27	4.46 ± 0.33	6.72 ± 0.46
T27	3.94 ± 0.27	3.00 ± 0.18	4.82 ± 0.36	5.48 ± 0.44
C1 (M1)	3.64 ± 0.24	3.20 ± 0.21	3.40 ± 0.20	4.08 ± 0.27
C2 (M2)	4.00 ± 0.29	2.92 ± 0.18	4.24 ± 0.28	4.26 ± 0.29
C3 (M3)	3.18 ± 0.19	4.96 ± 0.39	5.38 ± 0.35	5.96 ± 0.46

Values are presented as mean ± SD (*n* = 3). Exp: Experimental run; C: Uninoculated sludge control 1 (MIX 1); control 2 (MIX 2) control 3 (MIX 3).

**Table A7:** Measured MLVSS at 0 h, 6 h, 24 h and 48 h for B1, B2, and B3.

Exp	MLVSS, %			
	0 h	6 h	24 h	48 h
T1	64.81 ± 3.24	55.45 ± 2.77	79.28 ± 3.18	58.06 ± 2.74
T2	77.35 ± 1.83	55.59 ± 1.29	62.43 ± 1.88	82.09 ± 3.40
T3	78.55 ± 2.02	70.16 ± 1.35	67.15 ± 2.96	95.15 ± 1.07
T4	65.64 ± 3.28	93.65 ± 1.68	80.33 ± 3.62	64.34 ± 3.03
T5	76.46 ± 4.32	91.79 ± 2.57	90.41 ± 2.16	55.17 ± 2.57
T6	70.15 ± 3.23	90.91 ± 2.29	84.85 ± 2.62	87.57 ± 3.85
T7	73.15 ± 0.94	94.93 ± 4.53	71.17 ± 2.90	82.10 ± 1.69
T8	67.39 ± 2.31	89.84 ± 1.80	68.39 ± 1.41	45.20 ± 1.05
T9	76.93 ± 1.89	77.54 ± 3.64	78.57 ± 2.30	45.52 ± 1.30
T10	62.58 ± 3.13	88.21 ± 4.41	86.01 ± 3.78	62.00 ± 2.50
T11	85.48 ± 1.36	78.38 ± 1.63	75.68 ± 2.81	89.53 ± 2.82
T12	66.36 ± 3.26	70.00 ± 2.97	67.41 ± 2.51	89.29 ± 1.90
T13	57.14 ± 2.86	79.61 ± 3.98	82.86 ± 3.46	64.29 ± 2.93
T14	77.99 ± 3.90	70.98 ± 3.55	71.79 ± 2.97	58.08 ± 2.50
T15	77.10 ± 3.21	85.71 ± 2.72	64.81 ± 1.72	89.40 ± 4.01
T16	70.51 ± 0.85	86.54 ± 2.84	66.51 ± 1.14	77.34 ± 2.16
T17	74.62 ± 2.95	83.33 ± 3.98	95.43 ± 3.21	66.26 ± 0.79
T18	84.74 ± 3.89	78.32 ± 2.36	78.92 ± 2.89	87.97 ± 3.58
T19	69.11 ± 3.46	97.17 ± 0.91	71.31 ± 3.29	57.50 ± 2.54
T20	77.22 ± 3.49	76.71 ± 1.08	66.96 ± 1.12	76.75 ± 3.66
T21	79.82 ± 3.22	83.22 ± 3.26	73.80 ± 2.02	89.11 ± 0.91
T22	77.73 ± 3.89	88.15 ± 4.41	77.16 ± 3.19	57.55 ± 2.60
T23	81.90 ± 4.10	85.14 ± 4.26	67.40 ± 2.74	54.61 ± 2.27
T24	70.95 ± 3.10	90.43 ± 1.69	58.38 ± 1.22	84.51 ± 2.41
T25	72.12 ± 2.70	81.67 ± 1.54	67.71 ± 2.19	47.92 ± 2.01
T26	68.75 ± 2.82	86.62 ± 1.24	84.39 ± 2.00	82.87 ± 0.85
T27	79.80 ± 1.84	76.00 ± 0.88	66.49 ± 1.23	79.46 ± 0.94
C1 (M1)	70.45 ± 1.71	97.18 ± 0.82	84.17 ± 3.24	71.43 ± 2.53
C2 (M2)	80.00 ± 2.47	81.25 ± 1.10	72.22 ± 2.50	93.87 ± 3.34
C3 (M3)	66.06 ± 3.30	90.91 ± 4.55	74.89 ± 3.46	60.89 ± 2.72

Values are presented as mean ± SD (*n* = 3). Exp: Experimental run; C: Uninoculated sludge control 1 (MIX 1); control 2 (MIX 2) control 3 (MIX 3).

**Table A8:** ANOVA for the four RSM models based on the FFD.

Source	*SS*	d*f*	*MS*	*F*-Value	*p*-Value	
DCV—Reduced 2FI Model	671.28	9	74.59	7.79	0.0002	significant
A-OD	4.68	1	4.68	0.4886	0.4946	
B-Mix	373.28	2	186.64	19.49	<0.0001	
C-Bacteria	111.25	2	55.62	5.81	0.0127	
BC	185.17	4	46.29	4.84	0.0095	
Residual	153.19	16	9.57			
Cor Total	824.47	25				
DRV—Quadratic Model	5838.48	14	417.03	15.76	<0.0001	significant
A-OD	78.29	1	78.29	2.96	0.1111	
B-Mix	5393.19	2	2696.59	101.89	<0.0001	
C-Bacteria	133.84	2	66.92	2.53	0.1213	
AB	25.52	2	12.76	0.4821	0.6289	
AC	94.68	2	47.34	1.79	0.209	
BC	110.7	4	27.68	1.05	0.4241	
A^2^	2.26	1	2.26	0.0853	0.7753	
Residual	317.6	12	26.47			
Cor Total	6156.08	26				
FAV—Quadratic Model	10,329.37	14	737.81	21.61	<0.0001	significant
A-OD	100.59	1	100.59	2.95	0.1118	
B-Mix	10,007.46	2	5003.73	146.52	<0.0001	
C-Bacteria	8.95	2	4.48	0.1311	0.8784	
AB	123.77	2	61.88	1.81	0.2053	
AC	31.1	2	15.55	0.4554	0.6447	
BC	32.85	4	8.21	0.2405	0.9099	
A^2^	24.65	1	24.65	0.7218	0.4122	
Residual	409.8	12	34.15			
Cor Total	10,739.17	26				
REM—Quadratic Model	3775.28	14	269.66	52.35	<0.0001	significant
A-OD	155.35	1	155.35	30.16	0.0001	
B-Mix	3373.99	2	1687	327.5	<0.0001	
C-Bacteria	69.99	2	35	6.79	0.0106	
AB	109.38	2	54.69	10.62	0.0022	
AC	20.65	2	10.33	2	0.1773	
BC	45.83	4	11.46	2.22	0.1275	
A^2^	0.0848	1	0.0848	0.0165	0.9001	
Residual	61.81	12	5.15			
Cor Total	3837.09	26				

**Table A9:** Final predictive regression equations in actual terms for the four RSM models.

Response	Mix	Bacteria	Equations in Actual Terms
DCV removal, %	M1	B1	88.33 − 1.18∙OD
		B2	100.22 − 1.18∙OD
		B3	98.96 − 1.18∙OD
	M2	B1	90.85 − 1.18∙OD
		B2	88.74 − 1.18∙OD
		B3	95.11 − 1.18∙OD
	M3	B1, B2, B3	100.63 − 1.18∙OD
DRV removal, %	M1	B1	58.65 + 17.52∙OD − 3.07∙OD^2^
		B2	65.49 + 5.07∙OD − 3.07∙OD^2^
		B3	66.85 + 11.78∙OD − 3.07∙OD^2^
	M2	B1	49.62 + 11.08∙OD − 3.07∙OD^2^
		B2	47.53 − 1.36∙OD − 3.07∙OD^2^
		B3	47.09 + 5.34∙OD − 3.07∙OD^2^
	M3	B1	81.46 + 13.75∙OD − 3.07∙OD^2^
		B2	80.97 + 1.30∙OD − 3.07∙OD^2^
		B3	81.23 + 8.01∙OD − 3.07∙OD^2^
FAV removal, %	M1	B1	60.89 − 11.75∙OD + 10.16∙OD^2^
		B2	55.62 − 10.20∙OD + 10.16∙OD^2^
		B3	56.95 − 4.94∙OD + 10.15∙OD^2^
	M2	B1	57.85 − 0.77∙OD + 10.15∙OD^2^
		B2	57.26 + 0.77∙OD + 10.15∙OD^2^
		B3	52.80 + 6.04∙OD + 10.15∙OD^2^
	M3	B1	103.27 − 14.12∙OD + 10.15∙OD^2^
		B2	102.44 − 12.58∙OD + 10.15∙OD^2^
		B3	99.63 − 7.31∙OD + 10.15∙OD^2^
REM removal, %	M1	B1	80.90 + 3.89∙OD − 0.60∙OD^2^
		B2	77.74 + 6.90∙OD − 0.60∙OD^2^
		B3	72.69 + 9.71∙OD − 0.60∙OD^2^
	M2	B1	71.93 + 11.10∙OD − 0.60∙OD^2^
		B2	64.99 + 14.11∙OD − 0.60∙OD^2^
		B3	62.16 + 16.92∙OD − 0.60∙OD^2^
	M3	B1	101.47 − 2.28∙OD − 0.60∙OD^2^
		B2	99.86 + 0.73∙OD − 0.60∙OD^2^
		B3	98.36 + 3.54∙OD − 0.60∙OD^2^

Design-Expert 12 package was used for modeling of data.
